# Comparing Environmental Impacts of Single-Junction Silicon and Silicon/Perovskite Tandem Photovoltaics–A Prospective Life Cycle Assessment

**DOI:** 10.1021/acssuschemeng.4c01952

**Published:** 2024-05-23

**Authors:** Mitchell K. van der Hulst, Dorottya Magoss, Yiri Massop, Sjoerd Veenstra, Niels van Loon, Ilker Dogan, Gianluca Coletti, Mirjam Theelen, Selwyn Hoeks, Mark A. J. Huijbregts, Rosalie van Zelm, Mara Hauck

**Affiliations:** † Department of Environmental Science, Radboud Institute for Biological and Environmental Sciences, Radboud University, P.O. Box 9010, Nijmegen 6500 GL, The Netherlands; ‡ Expertise Group Circularity & Sustainability Impact, TNO, P.O. Box 80015, Utrecht 3508 TA, The Netherlands; § TNO Partner of Solliance, High Tech Campus 21, Eindhoven 5656 AE, The Netherlands; ∥ School of Photovoltaic and Renewable Energy Engineering, University of New South Wales, Sydney, NSW 2052, Australia; ⊥ FuturaSun Holding SRL, Riva del Pasubio 14, Cittadella (PD) 35013, Italy; # Technology, Innovation & Society, Department of Industrial Engineering & Innovation Sciences, Eindhoven University of Technology, P.O. Box 513, Eindhoven 5600 MB, The Netherlands

**Keywords:** life cycle assessment (LCA), prospective, ex-ante, emerging technology, passivated emitter and rear contact (PERC), multijunction PV, PV recycling

## Abstract

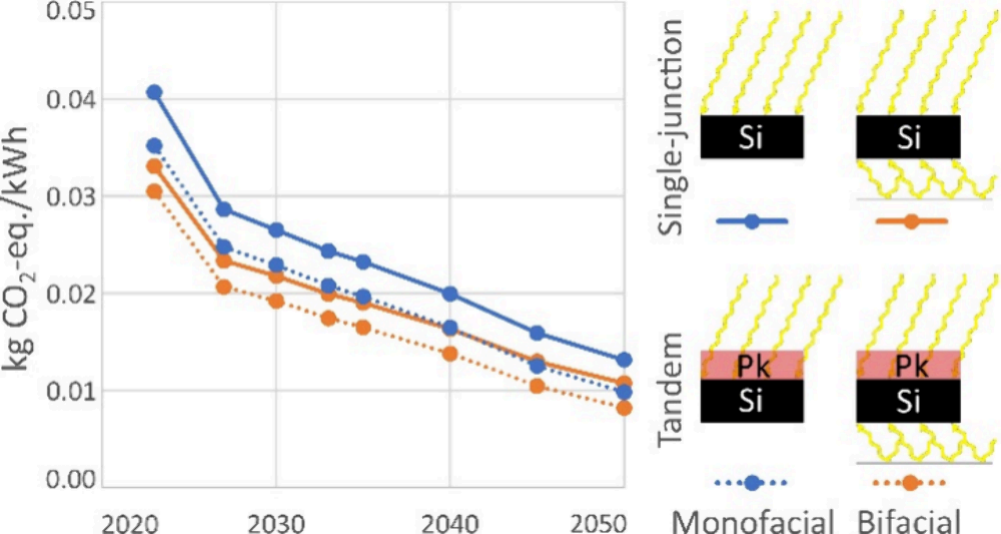

Tandem photovoltaics applying perovskite on silicon are considered to be a possible route to sustaining continuous efficiency improvements and price reductions. A meaningful market share for such tandems is, however, at least a decade away. Herein, a comprehensive prospective life cycle assessment was conducted, comparing the full life cycle of monofacial and bifacial silicon/perovskite tandem panels with single-junction silicon panels produced up to 2050. The end-of-life included the recovery of silicon and silver. Climate change impacts per kilowatt hour were projected to decrease by two-thirds over time. Tandem panels are expected to reach impacts of 8–10 g CO_2_-eq/kWh in 2050, while single-junction panels may reach 11–13 g CO_2_-eq/kWh in 2050. Other midpoint impact categories with substantial contributions to damaging human health and ecosystem quality were toxicity, particulate matter formation, and acidification, with tandems having lower impacts in each category. Reductions in impacts over time are mainly the result of grid mix decarbonization and panel efficiency improvements. Balance-of-system and recycling were found to contribute substantially to these impact categories. To ensure that tandem panels provide environmental benefits, annual degradation rates should not exceed 1% for monofacial or 3% for bifacial tandems, and refurbishment of panels with advanced degradation is crucial.

## Abstract 2

Environmental impacts of producing, using, and recycling single-junction silicon and silicon/perovskite tandem photovoltaics are predicted using prospective life cycle assessment.

## Introduction

Efficient and cost-effective photovoltaics (PVs) have become crucial in society’s effort to limit climate change. The dominant type of PV in the present-day market is single-junction silicon (i.e., solar cells made from a silicon semiconductor material).^1^ According to the Shockley–Queisser limit, the efficiency for single-junction PV is restricted to 33.77% for an ideal solar cell at a band gap of 1.34 eV.^2^ This limit can be exceeded by combining multiple semiconductor materials (i.e., multijunction PV) or by concentrating the sunlight. This concentrator PV can be a good option on the utility scale, but the need for lenses, mirrors, solar trackers, heat sinks, and/or cooling systems makes this option less suitable for residential application. This leaves multijunction PV as the most likely route for the broad application of high-efficiency PV.

Among multijunction PV, the combination of silicon and perovskite semiconductor materials in tandem provides multiple benefits: (1) expansive and expensive existing industrial-scale production capacity for silicon photovoltaics remains utilized, (2) the band gap of perovskites can easily be tuned to create an optimal match with silicon,^3^ and (3) materials used in silicon/perovskite tandems are cheaper and more abundantly available than the gallium and arsenic used in currently available high-performance multijunction photovoltaics.^4^ A recent prospective technoeconomic analysis demonstrated that adding perovskite to silicon PV adds less than 15% to the total cost of ownership.^5^ The increased efficiency results in silicon/perovskite tandem panels having a slightly lower levelized cost of electricity (LCOE) compared to single-junction panels.^5^ Furthermore, a larger decrease in LCOE was projected for the tandem than for the single-junction panel.^5^ Thus, silicon/perovskite tandem panels provide higher photovoltaic efficiency at a low additional cost, thereby reducing the overall cost per kWh.

Besides techno-economic prospects, environmental prospects are also important for making informed decisions on the further development and deployment of silicon/perovskite tandem photovoltaics. Life cycle assessment (LCA) has been used to evaluate whether the higher photovoltaic conversion efficiency of silicon/perovskite tandems outweighs the additional materials and energy required in production. Adding perovskite to silicon in a tandem device increases the environmental footprint per device by roughly 5 to 15% compared to the silicon-only option.^6,7^ In order to generate environmental benefits, this increase in environmental footprint per device needs to be overcompensated by an increase in electricity generated over its lifetime. Celik et al.^7^ found higher environmental impacts per kWh for silicon/perovskite tandem devices when compared to single-junction silicon devices due to a relatively low conversion efficiency and short perovskite lifetime included in their study. By assessing environmental impacts for varying lifetimes, Itten and Stucki^8^ found that the tandem needs to exceed a lifetime of 25 years to have a lower carbon footprint per kWh than the silicon-only option. Monteiro Lunardi et al.^9^ found similar results when assessing various silicon/perovskite tandem designs and highlight that not only the lifetime of the perovskite but also its transparency and conductivity at end-of-life are key characteristics for creating environmental benefits. It should be noted that all of the previously mentioned LCA studies were based on experimental laboratory data. The first LCA study for silicon/perovskite tandems produced in a pilot line was reported by de Wild-Scholten^10^ for the Oxford Photovoltaics Ltd. (OXPV) process. A more recent paper by Roffeis et al.^11^ contains an assessment of the same OXPV process on a “volume manufacturing line”. For the latter, it was also found that adding perovskite to silicon can provide environmental benefits, as long as lifetimes of the tandem devices are comparable to those of single-junction silicon devices.

As it will take several years for silicon/perovskite tandem PV to reach a substantial market share, it is important to assess future environmental impacts of this technology through prospective LCA. Of the mentioned LCA studies, only Itten and Stucki^8^ conducted a prospective assessment up to year 2025, with no other study assessing environmental impacts for longer time horizons. Furthermore, Itten and Stucki^8^ were the only ones to include the balance-of-system in their scope, while only de Wild-Scholten^10^ included a first-generation recycling process, with no study assessing the full life cycle of a complete, grid-connected PV system.

Here, we assess the current and future environmental impacts of the silicon/perovskite tandem PV panels compared to single-junction silicon PV panels over their full life cycle. We account for uncertainty regarding the stability of the perovskite by assessing a range of annual degradation rates and applying panel refurbishment for panels with short lifetimes. Furthermore, we account for technology developments in the foreground PV production system up to year 2050 and in the foreground PV recycling system up to year 2090 as well as developments in the background electricity, transport, fuels, steel, and cement systems up to year 2090. We consider both monofacial and bifacial designs, making it the first study to our knowledge to explicitly evaluate potential environmental benefits from bifaciality for silicon/perovskite tandems (i.e., capturing irradiation on both sides of the semiconductor material).

## Methods

This work follows the general workflow for LCA as outlined in ISO 14040,^12^ complemented by the framework for prospective LCA in van der Hulst et al.^13^ The goal and scope, life-cycle inventory (LCI), and life-cycle impact assessment (LCIA) are discussed here, while interpretation is reserved for the Results and Discussion section.

### Description of Studied PV Panels

**Figure 1:**
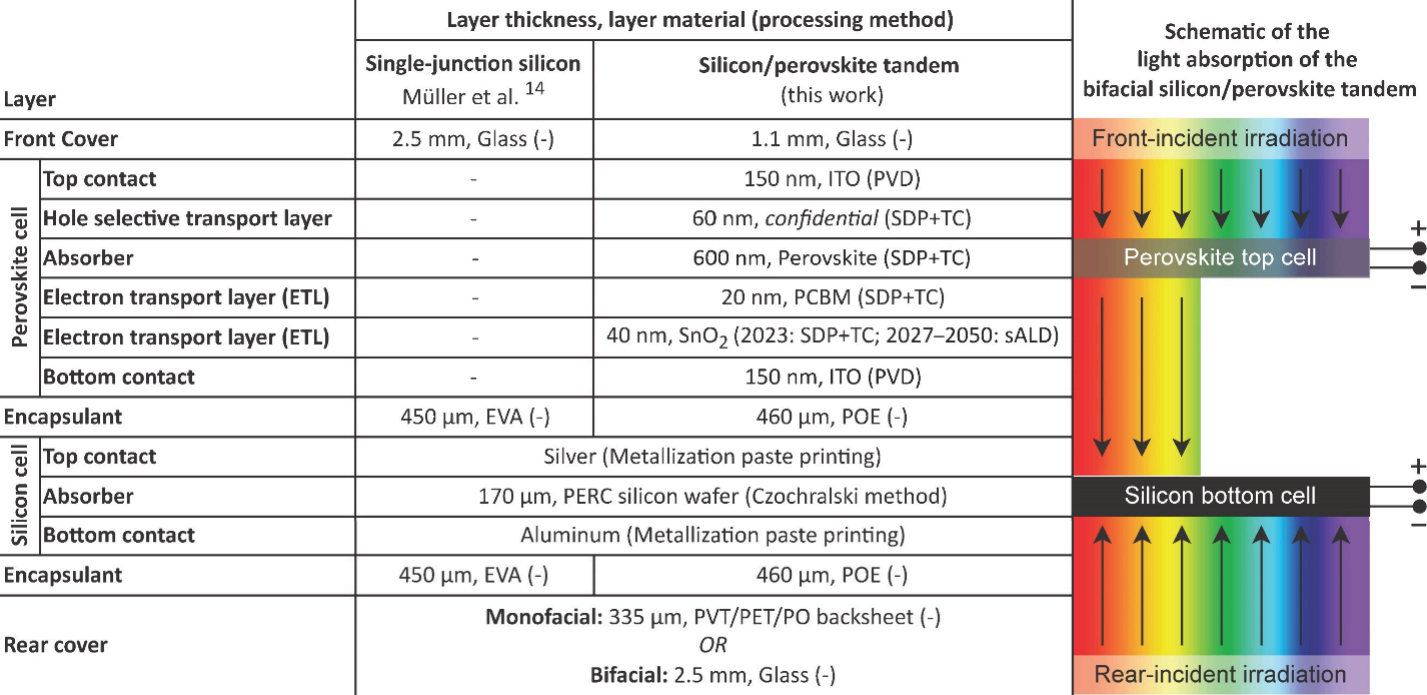
Configuration of the four product systems and schematic of the light absorption of the bifacial silicon/perovskite tandem panel. EVA, ethylene vinyl acetate; ITO, indium tin oxide; PCBM, [6,6]-phenyl C61 butyric acid methyl ester; PERC, passivated emitter and rear contact; PET, polyethylene terephthalate; POE, polyolefin elastomer; PVD, physical vapor deposition; PVT, polyvinyl toluene; sALD, spatial atomic layer deposition; SDP+TC, slot die printing and thermal cure; and SnO_2_, tin(IV) oxide.

All four assessed panels used PERC silicon cells. The tandem panels have a four-terminal (4T) design and employ an organometal lead halide perovskite layer. Figure 1 provides an overview of their configuration as well as a schematic of the light absorption of the bifacial silicon/perovskite tandem. Photons from front-incident irradiation and of energy below the perovskite band gap reach the silicon bottom cell and can be absorbed, while the entire photon spectrum of the rear-incident irradiation is absorbed by the silicon bottom cell.^14^ In monofacial panels, no rear-incident irradiation is absorbed due to the use of an opaque backsheet.

### Goal and Scope

The goal of the LCA is to compare the life-cycle environmental impacts of silicon/perovskite tandem PV panels to those of single-junction silicon PV panels. To compare the different types of panels, all results are reported relative to the same functional unit: “the provision of 1 kWh of electricity to the European Network of Transmission System Operators (ENTSO-E) grid”. The geographical scope of production is China, since this is where the majority of PV panels are currently produced,^1^ while the use and end-of-life phase were assumed to be in Europe.

**Figure 2:**
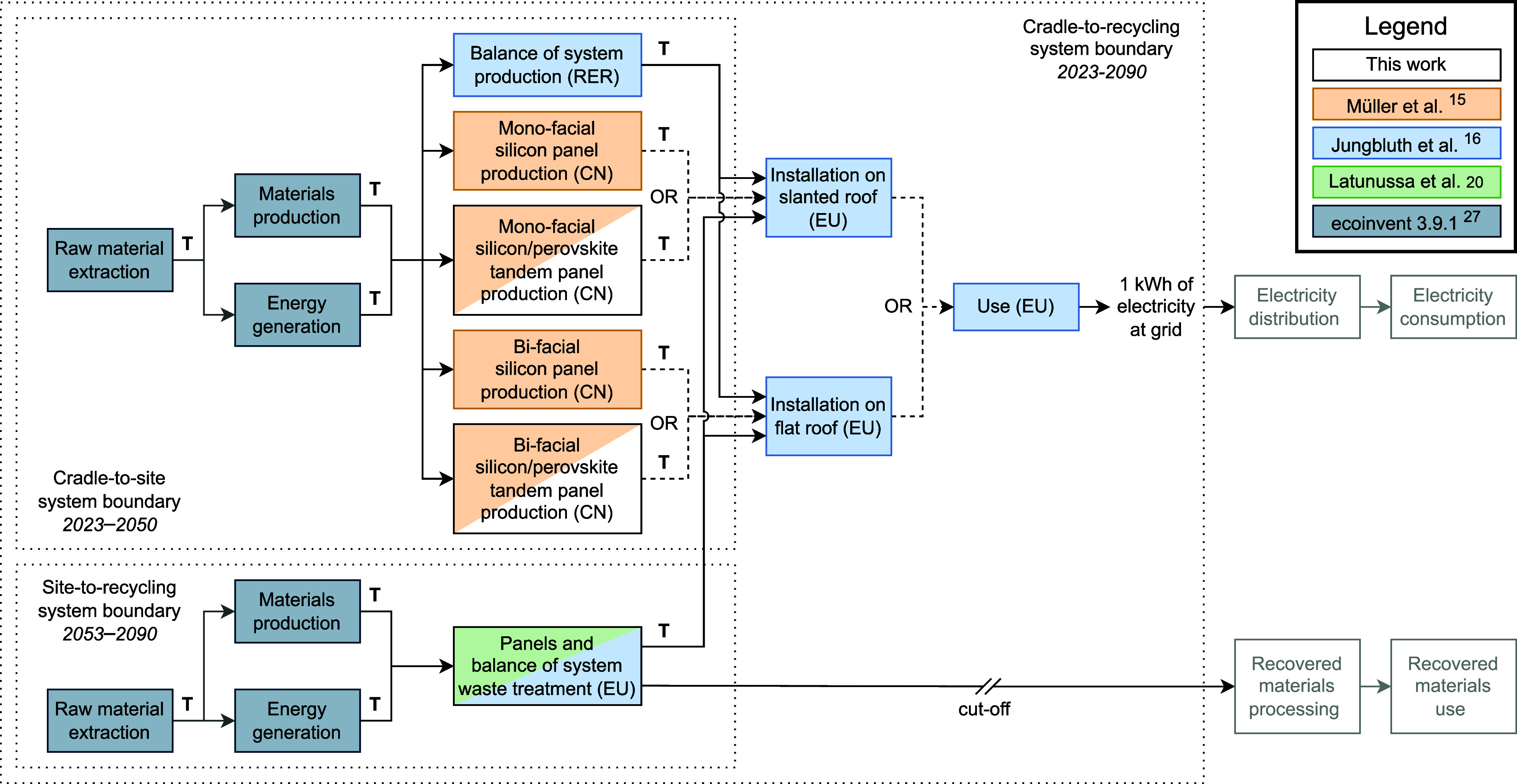
Flowchart for the product systems of this study, where either one of four panels is installed on either a slanted or flat roof, as indicated with dashed arrows. System boundaries are indicated by dotted lines. Materials recovered in waste treatment are cut off at the indicated point. (CN), process occurs in China; (EU), process occurs in Europe; T, transport.

The assessment followed the approach of an attributional LCA, since the aim was to evaluate the environmental footprint that can directly be associated with the electricity provided by the PV panels under the *ceteris paribus* assumption. Figure 2 provides a flowchart for the studied product system. Four panel types were studied, which were a monofacial and bifacial single-junction silicon panel and a monofacial and bifacial silicon/perovskite tandem panel. The monofacial glass backsheet panels were assumed to be framed and to be mounted on slanted roofs, whereas the bifacial glass–glass panels were assumed to be frameless and to be mounted on flat roofs. Distribution and use of electricity provided to the grid were placed outside the system boundary. For wastes, the cutoff system model was applied. Waste products are collected and disassembled at end-of-life, for which burdens were attributed to the waste producer, placing these processes inside the system boundary. Disassembly creates separate waste streams of recyclable products such as metals and glass. Burdens from processing these recyclables as well as benefits from the use of recyclables were attributed to the next life cycle, placing these processes outside the system boundary.

Data sources for life cycle inventories (LCIs) are indicated in Figure 2. LCIs for the industrial production of monofacial and bifacial single-junction silicon panels in China have 2020 as the reference year, making them the most recent LCIs for the industrial production of silicon PV to date.^15^ These LCIs were also used for monofacial and bifacial silicon/perovskite tandem module production, but with the front cover glass exchanged with a perovskite submodule based on in-house data from TNO. The resulting 4T silicon/perovskite tandem is comparable to the device presented in Coletti et al.,^14^ though the metal wrap through silicon heterojunction bottom cell was replaced with a passivated emitter and rear contact (PERC) silicon cell for lack of accurate LCI data. Detailed LCIs for the production of balance-of-system (BoS) components and maintenance were obtained from Jungbluth et al.^16^ The International Energy Agency Photovoltaic Power Systems Programme (IEA PVPS) recommends the use of a 30 year life expectancy for modules, transformers, and mounting systems and a 15 year life expectancy for inverters in the LCA of PV systems.^17^ However, lifetimes improve over time due to technological development. Therefore, we used projections from the Internal Technology Roadmap for Photovoltaics (ITRPV)^18^ for the life expectancies of modules (30–40 years) and inverters (12–18 years) and assumed that the life expectancy of transformers and mounting systems continues to match that of the module.

At end-of-life, panels and BoS were assumed to be fully recycled, in line with the European directive for waste electrical and electronic equipment.^19^ An LCI for the recovery of aluminum, glass, copper, silicon, and silver from waste panels was derived from Latunussa et al.,^20^ where process requirements were adjusted to the bill of material of the four product systems studied herein. This process is currently developed at the pilot/demonstration plant level^21^ and was assumed to be operational at an industrial level in 2053 when panels produced in 2023 reach their end-of-life. The LCIs of all foreground processes are provided in Supporting Information 2 and their construction is described in further detail in Supporting Information 1, section 1.

At present, single-junction silicon PV panels have a lifetime of 30 years, which is projected to increase to 40 years in 2030, mainly by improving the properties in encapsulation and backsheet materials to ensure long-term stability.^18^ For tandem panels to match such lifetimes, refurbishment is required when annual degradation rates substantially exceed those of single-junction devices due to instability of the perovskite material.^22^ The considerable time lag between production and end-of-life should be accounted for in the prospective assessment by modeling the waste treatment processes at the moment of end-of-life to prevent over- or underestimating future burdens from the delayed waste treatment process.^23^ Panels produced in 2023 have a use phase spanning 2023 to 2053, while panels produced in 2050 have a use phase spanning 2050 to 2090. Thus, the temporal scope of the study is 2023 to 2090, with the temporal scope of the cradle-to-site system being 2023 to 2050 and the temporal scope of the site-to-recycling system being 2053 to 2090. The shorter life expectancy of inverters compared to panels was accounted for by including the use of multiple inverters over the lifetime of the panel, with the production of each inverter modeled in the same years as the production of the panel for simplicity.

### Electricity Generation

Environmental impacts from solar panel production and waste treatment were divided by the lifetime electricity output *E*_*LT*_ [kWh/m^2^] of the panel to express the impact per functional unit (i.e., per kWh). This lifetime electricity production depends on several system parameters such as the type (single-junction or tandem), design (monofacial or bifacial), efficiency, and annual degradation rate. Furthermore, the lifetime yield is affected by location-dependent parameters such as the insolation at the site of use and the performance ratio which were kept constant in this study. The lifetime electricity for a panel of a specific type and design was calculated using eq 1, with values for the variables reported in Table 1. The assumed insolation *I* is the population-weighted average for the European Union as also used by Müller et al.,^15^ and the performance ratio *PR* is that of a typical present-day system. The initial power density *PD*_*i*_ is a measure of the solar panels’ ability to convert the energy of sunlight to electricity. While normally expressed in W/m^2^ relative to front illumination, *PD*_*i*_ is herein expressed as a percentage by normalizing it to 1 sun (i.e., 1000 W/m^2^ front illumination). For the bifacial panels, an additional 200 W/m^2^ rear-incident irradiation is assumed, in line with BiFi200 test conditions. This additional irradiation results in an increased electricity output for the bifacial panel, which is expressed as a percentage relative to that of its monofacial counterpart. For the single-junction panel, this monofacial/bifacial power density ratio is assumed to be 117.5%, while for the tandem panel it is slightly lower at 115%. These ratios were assumed to remain constant over time and were derived from power densities reported by Coletti et al.^14^ for single-junction and tandem panels under BiFi0 and BiFi200 conditions. Initial power densities for the tandem panel decrease when going from 2023 to 2027, which is typical when going from small-scale to industrial-scale panels. Finally, the sum function of eq 1 was used to correct for the annual degradation rate *DR* of the solar panel over its lifetime *LT*, which was assumed to remain constant beyond 2033.
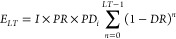
1

**Table 1:** Assumed Values for the Performance Parameters in Equation 1 Used to Calculate the Lifetime Electricity Output

	System	Unit	2023	2027	2030	2033	2035	2040	2045	2050	Source
*I*	All	kWh/m^2^/year	1391								(24)
*PR*	All	%	83								(1)
*PD*_*i*_	Single junction, monofacial	%	21.4	22.0	22.5	22.8	23.0	23.5	23.8	24.0	‘23–‘33,^18^ ‘35-‘50: extrapolation
	Single junction, bifacial	%	25.1	25.9	26.4	26.8	27.0	27.6	28.0	28.2	117.5% of single-junction, monofacial
	Tandem, monofacial	%	26.5	26.1	26.7	27.3	27.7	28.7	29.7	30.7	‘23,^14^ ‘27–‘33,^18^
											‘35–‘50: extrapolation
	Tandem, bifacial	%	30.5	30.0	30.7	31.4	31.9	33.0	34.2	35.3	115% of tandem, monofacial
*LT*	All	year	30	32.5	40						(18)
*DR*	Single junction	%	0.5	0.45	0.4						(18)
	Tandem	%	0.5 or 1 or 2 or 3								scenario analysis

### Scenario Analysis for the Annual Degradation Rate

For the tandem systems, stability of the perovskite is one of the current concerns hindering commercialization. The record for sustained performance of a perovskite device is in the order of months, not decades.^25^ In order for the tandem panels to be economically viable, their lifetime electricity output should exceed that of conventional single-junction panels. Herein, we assessed the scenario where tandem panels are removed from the field for refurbishment once their annual electricity output drops below that of a comparable single-junction device with the same lifetime. Tian et al.^22^ found that, with refurbishment, degradation rates for silicon/perovskite tandems should not exceed 3% (relative) from the outset to ensure that greenhouse gas footprints are lower than those of single-junction silicon. To assess the sensitivity of results for the assumption of annual degradation rates for the tandem system, these were varied from 0.5 to 3% (relative), in line with observations for systems installed in the past decade.^26^ Because of the lack of insights into the exact developments of the efficiency of the top and bottom cells, the power density of the refurbished panel was assumed to be the same as its initial power density. To model the refurbishment process, the panel was assumed to be transported to a refurbishment facility where the frame and wiring are cut off and where the perovskite submodule is removed by heating the encapsulant. A new perovskite submodule is placed on top of the existing bottom module using a new encapsulant and edge seal. The panel is finished with wiring and a frame and transported back to the site. The tandem panel is assumed to reach its end-of-life at the same time as its single-junction counterpart (i.e., after 30 to 40 years). For high annual degradation rates, this may result in the tandem panel being refurbished up to four times over its lifetime, which is the upper limit for the number of cycles considered by Tian et al.^22^ It should be noted that materials required for refurbishment were modeled to be produced in the same year as the panel so as not to overcomplicate the model. In section 2 of Supporting Information 1, a visual representation is provided of lifetime electricity outputs for tandems with high annual degradation rates that are refurbished.

### Prospective Assessment

Future developments in the foreground system were identified using the framework for prospective LCA in van der Hulst et al.^13^ A detailed account of its application is provided in Supporting Information 1, section 4. First, the technological and manufacturing readiness levels of each system component were assessed. Tandem production and recycling with silicon and silver recovery have not yet been applied at a commercial level. Modeling these processes requires the inclusion of developments toward technology and manufacturing readiness. For the tandem production, these developments were derived from interviews with technology experts at TNO, while for recycling these developments were derived from Frelp by Sun.^21^ Changes in tandem production relate to size scaling as well as process changes that improve the coverage and mechanical and thermal stability of the electron transport layer and utilize safer solvents in slot die printing. Changes in the architecture of the tandem (i.e., alternative materials or layer orders) were not modeled due to the myriad combinations involved.

Additional developments for industrially produced technologies were also assessed. Industrial learning in the foreground system was derived from the most recent version of the ITRPV^18^ and translated into changes in silicon consumption, efficiencies, panel and BoS lifetimes, and degradation up to year 2033. Silicon consumption and efficiency were expected to continue improving and were therefore further extrapolated up to year 2050, while the annual degradation rate and by extension the panel lifetime as well as BoS lifetimes were assumed to stabilize at the 2033 level and were therefore kept constant up to year 2050.

The biosphere and background systems of the technosphere were represented by the databases biosphere3 and ecoinvent, version 3.9.1, system model “Allocation, cut-off by classification”,^27,28^ respectively. For the background system, developments in the sectors of electricity generation, transport by truck, and production of cement, steel, and fuels were modeled using the python package premise, version 2.0.2.^29,30^ Premise takes the ecoinvent database as input and adds several data sets for new technologies in various markets, for example, a coal-fired power plant with carbon capture and storage. By applying premise, copies were made of the ecoinvent LCI database, and various data sets in this database were adapted to represent a future state. Subsequently, these copies of ecoinvent were combined in a superstructure database and scenario difference file to streamline further scenario analyses.^31^ Developments were based on projections from the integrated assessment model IMAGE.^32^ The selected projections follow shared socioeconomic pathway 2 (SSP2), known as the “middle of the road” pathway, which assumes that social, economic, and technological developments follow a similar trajectory to that charted in the past. Its baseline scenario results in a global mean surface temperature increase of 3–4 °C by 2100. These projections were further differentiated with representative concentration pathways (RCPs) which project the final radiative forcing from greenhouse gases that is reached by year 2100.^33^ Herein, the RCP 2.6 and 1.9 scenarios were assessed, which coincide with radiative forcings of 2.6 and 1.9 W/m^2^, resulting in global mean surface temperature increases of 1.6–1.8 and 1.2–1.4 °C by 2100, respectively.

### Life Cycle Impact Assessment

Impacts were assessed following the ReCiPe 2016 method.^34^ Impacts were assessed for the 22 midpoint impact categories in ReCiPe 2016 as well as the three endpoint impact categories. The latter were used for the broadness of their scope, enabling an assessment of various midpoint impact categories with divergent units and their net effects on the three areas of protection: human health, ecosystem quality, and resource scarcity. Results at the endpoint level are expressed in terms of damage to human health, in disability-adjusted loss of life years (DALY), damage to ecosystem quality, expressed in time-integrated species loss (species·year), and damage to resource availability, expressed in surplus cost (USD2013).^34^ A midpoint to endpoint contribution analysis was conducted to identify which of the 22 midpoint categories are the most relevant damage pathways. Special focus was placed on the climate change midpoint category for its policy relevance and because it is commonplace in LCAs of solar panels, with impacts expressed in kg CO_2_-eq. Further note that the ReCiPe 2016 method allows for the comparison of results with the study from Roffeis et al.,^11^ which is the only study to date to assess environmental impacts of an industrially manufactured silicon/perovskite tandem.

The ReCiPe 2016 method considers three scenarios or perspectives based on culture theory. The results presented in the next sections follow the hierarchist (H) perspective, which considers time frames and available data for which there is scientific consensus.^35^ For the midpoint of climate change, this means that the global warming potential for a 100 year time horizon (GWP100a) was considered. In Supporting Information 1 and 2, results for the other two perspectives are also provided. These are the individualist (I) perspective, which “is based on the short-term interest, impact types that are undisputed, and technological optimism with regard to human adaptation”, and the egalitarian (E) perspective, which “is the most precautionary perspective, taking into account the longest time frame and all impact pathways for which data are available”.^35^

To enable cross-study comparability with other LCA studies for PV systems, we also used the set of impact assessment methods recommended by the IEA PVPS.^17^ This set consists of the categories contained in the environmental footprint (EF) v3.1 method,^36^ supplemented with the cumulative energy demand (CED)^37^ and an indicator for the biodiversity damage potential caused by land use. For the latter, we used the land use indicator from the ReCiPe endpoint (H) since it is the only method available in the used software that expresses land use impacts in terms of biodiversity loss as recommended by IEA PVPS. The additional recommended indicator of nuclear waste was disregarded for lack of a clear link between the reported characterization factors and the ecoinvent inventory database.

Impacts per unit process were calculated using Activity Browser version 2.9.7,^38,39^ which provides a graphical user interface to the python-based LCA software Brightway2.^40^ The climate change category in the default ReCiPe 2016 and EF v3.1 impact assessment methods was adapted to account for negative emissions in technologies such as bioenergy with carbon capture and storage (BECCS) and emissions of hydrogen, as is recommended for databases adapted with premise (see Supporting Information 1, section 4).

### Variance Decomposition Analysis

A variance decomposition analysis was conducted to assess to what extent the results were affected by the following sources of variance: initial panel efficiency (Table 1, section (2), background scenario (SSP2 – baseline, SSP2 – RCP2.6, and SSP2 – RCP1.9), panel design (mono- or bifacial), and annual degradation rate (0.5, 1, 2, or 3% (relative); only for tandem panels). We performed one variance decomposition analysis per combination of the panel type and impact category in order to derive the sum of squares for all potential sources of variance. For each panel type and impact category, the obtained sum of squares for each of the sources of variance was divided by the total sum of squares, resulting in the relative variance attributable to each source.

## Results and Discussion

### End Point

**Figure 3:**
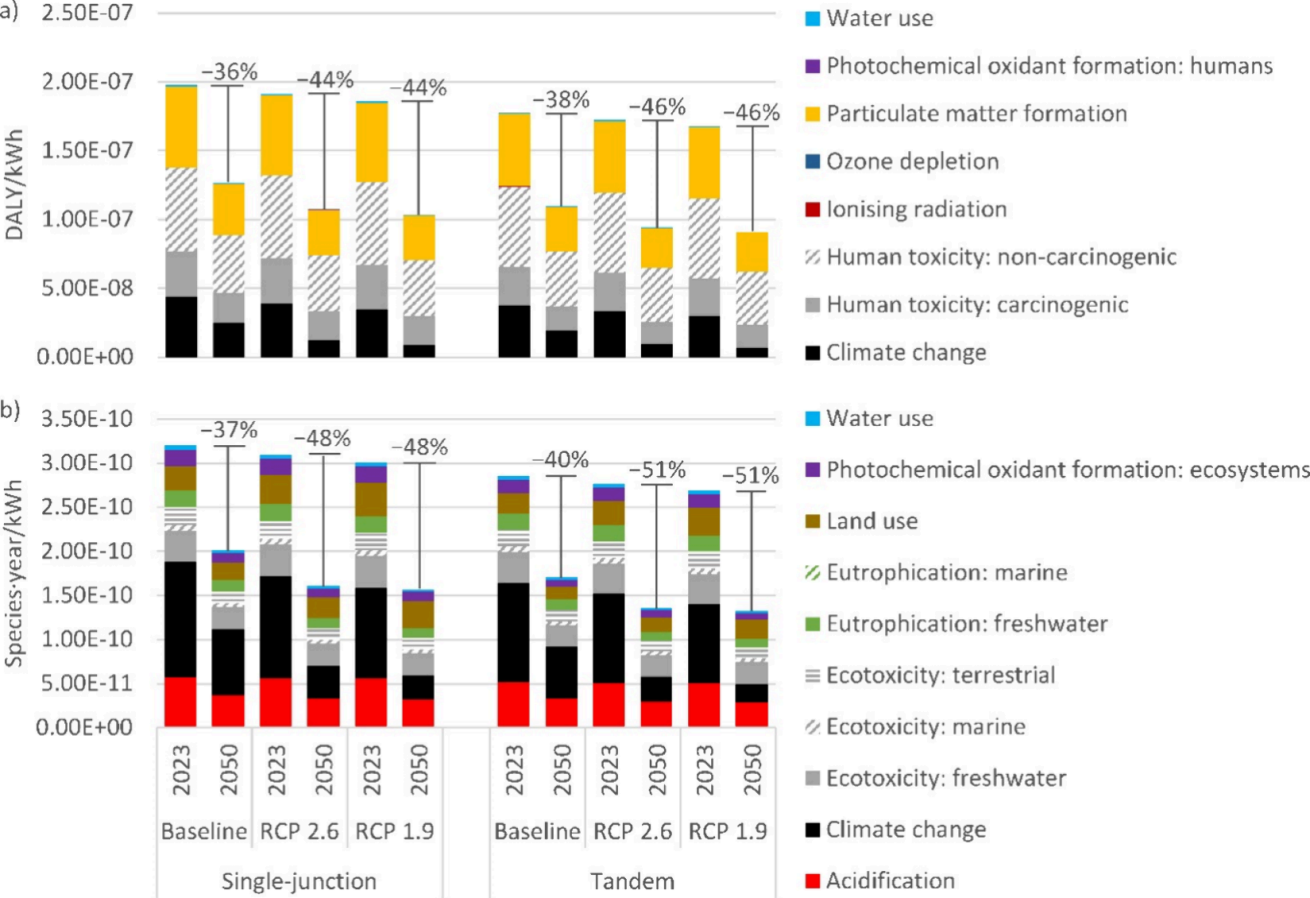
Midpoint to endpoint contribution analysis for the endpoints of (a) human health in DALY/kWh and (b) ecosystem quality in species·year/kWh using the hierarchist (H) perspective of the ReCiPe 2016 LCIA method. Percentages represent the decrease in impact between 2023 and 2050 for each respective background scenario (i.e., shared socioeconomic pathway 2 (SSP2) – baseline, SSP2 – representative concentration pathway (RCP) 2.6, and SSP2 – RCP 1.9, corresponding to 3–4, 1.6–1.8, and 1.2–1.4 °C global mean surface temperature increases by 2100, respectively). Other assumptions: panel design, monofacial; insolation, 1391 kWh/m^2^/year; geographic scope, China (production) and Europe (use and end-of-life); annual degradation rate for perovskite, 0.4–0.5% (relative).

Impacts to the human health and ecosystem quality areas of protection were projected to decrease by one-third between 2023 and 2050 for the SSP2-based scenario and by about half for the SSP2-RCP2.6 and SSP2-RCP1.9 scenarios (Figure 3). Results for the resource scarcity area of protection show similar trends and are provided in Supporting Information 1, section 5.1. When looking at individual midpoint to endpoint contributions, we find that climate change is the major contributor to the ecosystem quality endpoint category and a substantial contributor to the human health endpoint (Figure 3). Climate change impacts decrease more sharply than the overall endpoint categories of human health and ecosystem quality, thus an improvement in this midpoint category is a major contributor to projected improvements in these endpoint categories. Other relevant midpoint categories with contributions above 15% were toxicity, particulate matter formation, and acidification (Figure 3). These categories should therefore be explicitly included in the environmental impact assessment of single-junction silicon and silicon/perovskite tandem systems rather than focusing solely on climate change. Of lower priority are water and land use, photochemical oxidant formation, and freshwater eutrophication (Figure 3). Of lowest priority are the categories of marine eutrophication, ionizing radiation, and ozone depletion with contributions below 1% (Figure 3).

Results in Figure 3 were generated for the hierarchist (H) perspective of the ReCiPe 2016 life cycle impact assessment model, while the individualist (I) and egalitarian (E) perspectives are presented in Supporting Information 1, sections 5.2 to 5.6. What stands out is the large contribution of the human toxicity impact categories in the egalitarian perspective. Emissions of chromium and zinc in copper and steel production for balance-of-system components play a major role, since characterization factors for these elementary flows are, respectively, 2 and 4 orders of magnitude larger in the egalitarian perspective.

### Midpoint – Climate Change

**Figure 4:**
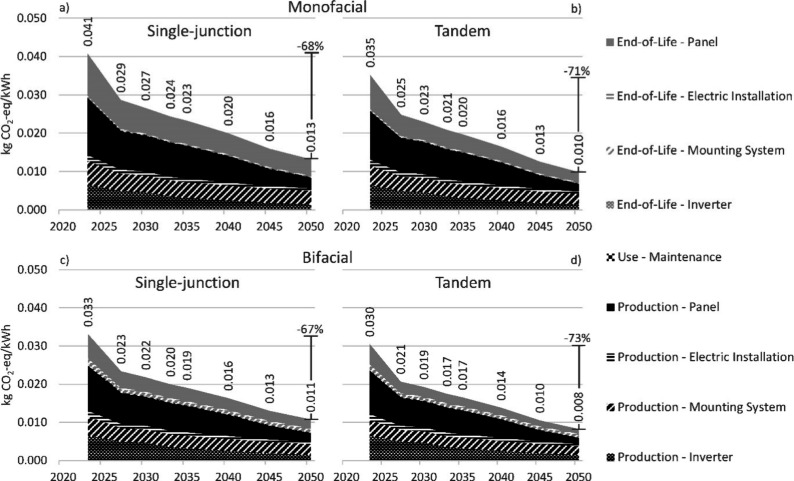
Process contribution analysis for the impact category of climate change in kg of CO_2_-eq/kWh using the EF v3.1 method. The *x* axis represents the year of production for the panel. Impacts from end-of-life were modeled to occur after the economic lifespan of the panel. For example, end-of-life for the panel produced in 2023 was modeled to occur in 2053. Assumptions: panel design: (a) monofacial single-junction, (b) monofacial tandem, (c) bifacial single-junction, or (d) bifacial tandem; insolation: 1391 kWh/m^2^/year; geographic scope: China (production) and Europe (use and end-of-life); annual degradation rate for perovskite: 0.5% (relative); background scenario: shared socio-economic pathway 2 - representative concentration pathway 2.6 (corresponding to a 1.6–1.8 °C global mean surface temperature increase by 2100).

Figure 4 shows that the climate change impact per kWh decreases over time by around two-thirds as a result of all developments included in the prospective assessment. Displayed results are for the EF v3.1 method and the SSP2-RCP2.6 scenario (i.e., when policy is enacted to limit global warming to 1.6–1.8 °C). Climate change impacts are less than halved in the SSP2-base scenario, while the more stringent SSP2-RCP1.9 scenario results in reductions of almost three-quarters (Supporting Information 1, sections 5.7 and 5.8). When investigating design choices in Figure 4, we see that climate change impacts reduce (1) by −15 to −23% when adding perovskite to single-junction silicon, (2) by −15 to −20% when making single-junction silicon panels bifacial, and (3) by −27 to −38% when combing both options to create a bifacial tandem panel. The climate change category of EF v3.1 uses characterization factors from the recent sixth assessment report of the IPCC,^42^ while ReCiPe 2016 uses outdated characterization factors from the fifth assessment report,^43^ resulting in slightly lower (2–4%) results when using the EF v3.1 method. For climate change results of all perspectives of the ReCiPe 2016 method, see Supporting Information 1, sections 5.9 to 5.17.

The main contributors to the climate change impact are the production and end-of-life waste treatment of the panel. Production requires a substantial amount of process energy, in particular, in the supply chain of the silicon wafer, with emissions from energy generation contributing to climate change. Reductions of climate change impacts per kWh over time are the result of increased panel efficiency in combination with decarbonization of the energy sector. In end-of-life waste treatment, large amounts of liquid waste and sludge are produced in acid leaching and subsequent electrolysis of the bottom ash for the recovery of silicon and silver. Emissions from combustion engines during the transport of these waste streams contribute to climate change, with observed reductions in climate change impacts per kWh being the result of increased displacement of fossil fuels in transport as well as increased panel efficiency, which reduces the amount of waste panel on a per kilowatt hour basis. It should be noted that the modeled waste treatment is in an early stage of development and therefore contains more uncertainty than the modeled production processes.

The considerable contribution of end-of-life waste treatment of the panel is striking, since dismantling and recycling contributed only 2–3% to the climate change impact results in de Wild-Scholten.^10^ They used LCI data provided by IEA PVPS for first-generation recycling which recovers bulk materials only.^41^ Such recycling schemes are in line with the target set out in the European waste directive to recover 85 wt % of the PV waste.^19^ In this work, however, we explicitly modeled a more extensive recycling process since there are multiple incentives to also recover silicon and silver, such as more stringent circularity objectives, concerns over material scarcity, and the desire to reduce the dependence on imports. The considerable contribution of such waste treatment highlights the importance of including this life-cycle stage in prospective LCA rather than excluding it for the lack of data on mature recycling schemes. Close collaboration between the research communities for PV production and recycling is desirable to devise strategies that minimize environmental impacts over the entire life cycle of the PV panel (i.e., by producing panels in a way that simplifies silicon and silver recovery, circumventing wasteful leaching and electrolysis processes).

This work explicitly accounted for the time lag between the production and end-of-life waste treatment. For example, results for 2023 include the production of a panel in 2023 and end-of-life waste treatment in 2053. End-of-life waste treatment was therefore modeled with a greener electricity mix than production. Section 5.18 of Supporting Information 1 displays results for the impact category of climate change when not accounting for time lag (i.e., placing production and end-of-life at the same point in time). The climate change impacts were found to be overestimated by up to +23%, highlighting the importance of avoiding a temporal mismatch between the foreground and background systems.

The lifetime of the silicon-perovskite tandem presented a large uncertainty within this study. Previous studies already indicated that tandem devices with perovskite require a lifetime of over 20 years to have an equivalent environmental performance when compared to their single-junction counterpart.^8,9,11^ In Figure 4, the stability of the perovskite was assumed to be comparable to that of single-junction silicon panels (i.e., 0.4–0.5% (relative) annual degradation and lifetimes of 30 to 40 years). Impacts from the use phase are negligible since no refurbishment is required. However, substantial contributions from the use phase are observed at higher annual degradation rates (see Supporting Information 1, section 5.19). As a result, at annual degradation rates above 1% (relative), monofacial tandem panels have a higher climate change impact per kWh than monofacial single-junction panels (see Supporting Information, section 5.20). Comparing bifacial panels, tandems were found to have a lower climate change impact per kWh than single-junction panels at annual degradation rates of up to 3% (relative; see Supporting Information 1, section 5.20). Thus, while present-day annual degradation rates for perovskite exceed 3% (relative), annual degradation should not exceed 1% (relative) in the case of monofacial panels and should not exceed 3% (relative) in the case of bifacial panels for tandem devices to have lower climate change impacts than single-junction devices. Such degradation rates are within the range of rates observed in the field for competing commercial products.^26^

### Midpoint – Other Categories

Results for all midpoint impact categories of the ReCiPe 2016 method and the set of methods prescribed by the IEA PVPS are provided in Supporting Information 2. Here, findings for midpoint categories with substantial contributions to the three endpoints are discussed, which are the midpoints of toxicity, particulate matter formation, and acidification. Using the Sankey diagram feature of Activity Browser 2.9.7, contributions to these midpoints were traced back to smelting processes in the production of copper and aluminum used in the balance of the system. During the smelting process, toxic heavy metals vaporize from the ore, of which part condenses to form particulate matter. In addition, sulfur dioxide is emitted during the smelting processes, contributing to acidification. These results emphasize that while outside the sphere of influence for PV panel developers, the inclusion of balance-of-system is crucial in getting a representative picture of the total life cycle environmental impacts related to electricity from solar panels.

For the toxicity categories, it should be noted that equivalent contributions are observed for the single-junction and tandem devices (Figure 3), indicating that lead in perovskite is not a major contributor to toxicity. This is in line with earlier finding that suggest that lead does not make a substantial contribution to the impact categories of human toxicity, despite continued concerns.^44,45^ However, this does not mean that such concerns are unwarranted. While lead emissions per kWh of electricity are insignificant, such a finding does not account for scenarios where exposure to lead can be high, such as in occupational exposure for factory workers or in incidental exposure from a broken panel due to extreme weather events or fire.^44^ Risk assessment would be more suitable to assess such scenarios, as, for instance, conducted by Blanco et al.^46^ for arsenic, gallium, and indium in III–V/silicon tandem solar cells.

When midpoint results were compared between ReCiPe 2016 and EF v3.1, general trends held. Both methods show that impacts decrease over time, that tandems have lower footprints than single-junction panels, and that bifacial panels have lower footprints than monofacial panels.

### Variance Decomposition Analysis

**Figure 5:**
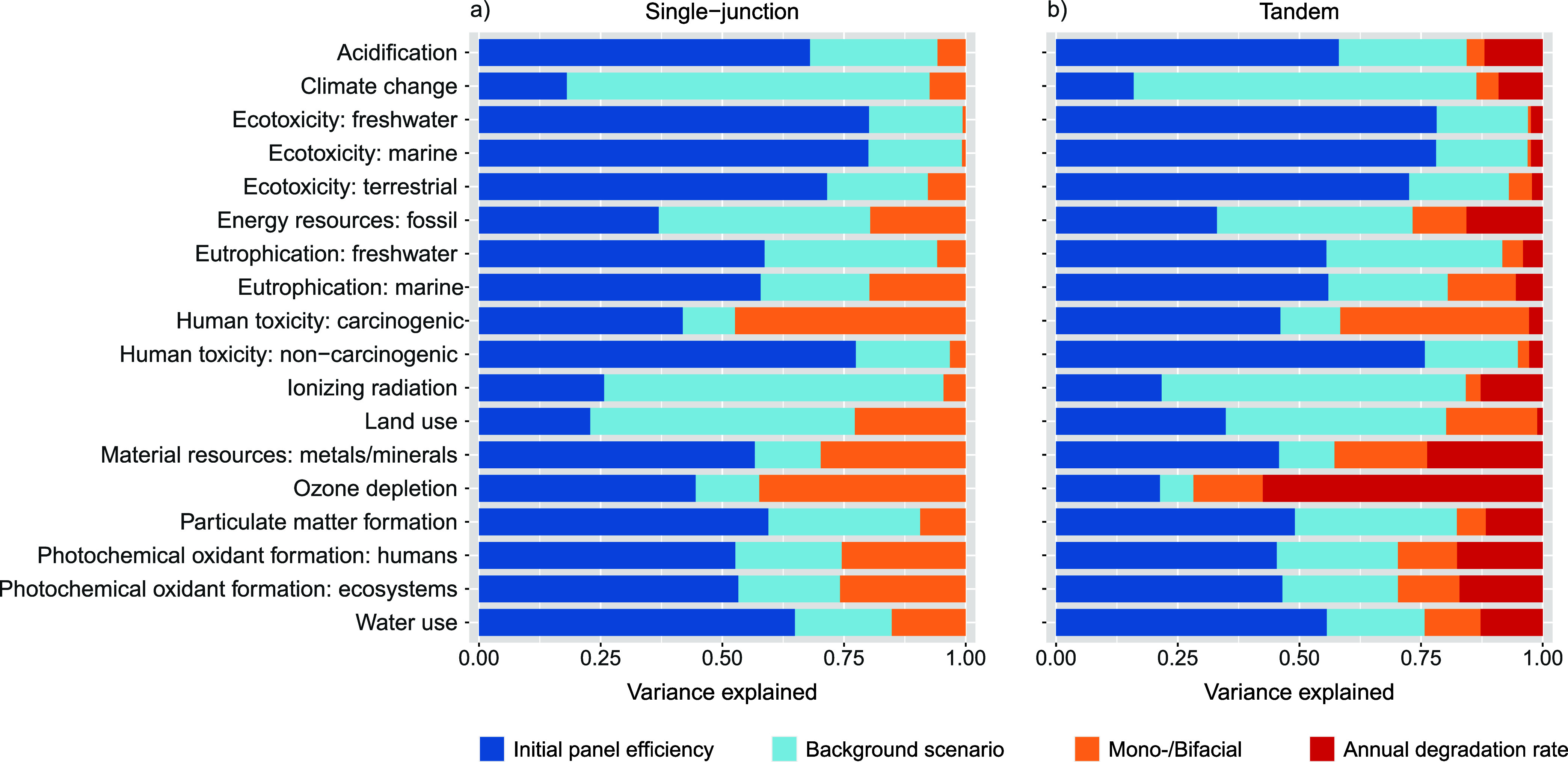
Variance decomposition analysis for the results of all midpoint impact categories for (a) the single-junction panel or (b) the tandem panel using the hierarchist (H) perspective of the ReCiPe 2016 LCIA method.

Figure 5 presents the results for the variance decomposition analysis of footprints calculated with the ReCiPe 2016 (H) method. Results for the set of impact categories recommended by the IEA PVPS are provided in Supporting Information 1, section 5.21. Each of the four variables assessed was found to be an important source of variance in at least one of the impact categories. For a variable to be an important source of variance, it needs to have a strong influence on the total environmental impact or it needs to be highly variable or be both influential and variable. Figure 5 reveals that the initial panel efficiency explains more than half of the variance in 12 of the 18 midpoint impact categories for the single-junction panel. This is to be expected since the efficiency has a strong influence on the lifetime electric output, with higher outputs resulting in lower impacts per functional unit (i.e., per kWh provided to the ENTSO-E grid). Efficiency is also highly variable, ranging from 21.4 to 35.3%, as reported in Table 1.

The background scenario was found to be the major contributor to the variance in impact categories which are strongly influenced by emissions in electricity generation, transport, and production of fuels, steel, and cement. These were the categories of climate change, fossil energy resources, ionizing radiation, and land use (Figure 5). Climate change and fossil resources are affected by changes in the amount of fossil fuels combusted for energy generation, transport, and production of a wide range of material. Land use is affected by a shift in the transportation sector from fossil fuels to biofuels. Ionizing radiation is affected by an increased deployment of nuclear energy in scenarios with greener energy mixes. To our knowledge, only one other prospective LCA study on silicon/perovskite PV has been conducted,^8^ but this study did not include such external developments, thereby likely overestimating future footprints in these categories.

The panel design (i.e., whether the panel is monofacial or bifacial) is the major contributor to the variance in results for the impact category of human carcinogenic toxicity (Figure 5). Residual landfilling of bottom ash from panel waste treatment has a strong influence on this category. The bifacial panel utilizes two glass panes as front and back covers, whereas the monofacial panel utilizes glass only as the front cover and a thin plastic sheet as the back cover material. As a result, the amount of waste going to residual landfilling is highly variable, thus explaining why mono/bifaciality is an important contributor to the variance in this impact category.

The degradation rate contributes only to the variance of the tandem panel since a single value was assumed for the single-junction panels. It was found to be a major contributor to the variance in the category of ozone depletion (Figure 5). The additional processing for panel refurbishment requires additional perovskite material, with chloromethane emissions during the production of the confidential layers contributing strongly to the midpoint category of ozone depletion. A scenario with no refurbishment was not assessed since it is unlikely that tandems could financially compete with single-junction panels under such conditions. However, in such a scenario, it is to be expected that the degradation rate would be the dominant variable contributing to variance in all impact categories since it would strongly influence the lifetime electricity output.

### Comparison to Literature

The results from our study were compared to de Wild-Scholten^10^ and Roffeis et al.,^11^ who studied the production of silicon/perovskite tandem panels at near-industrial and industrial scales, respectively. Direct comparison is complicated due to differences in scope and several assumptions. For example, Roffeis et al.^11^ consider only the production of the panel, thus excluding impacts from the production of the BoS and the use and end-of-life of both the panel and BoS. de Wild-Scholten^10^ did consider a similar scope to our study but assumed a first generation recycling process that excludes the recovery of silicon and silver, thereby excluding most of the impacts from end-of-life waste treatment as already mentioned. Neither study was prospective, thus improvements due to technological developments were not considered. Both studies made different assumptions in terms of panel efficiency, performance ratio, lifetime, degradation rate, and insolation. Correcting for these differences, we find our reported environmental impacts to be of the same order of magnitude. Furthermore, the studies are in agreement about tandem panels providing electricity with a lower environmental impact per kWh than single-junction panels. At the level of individual impact categories, results vary widely between the two studies and between these studies and our work, both when looking at the total impact per kWh and when looking at the individual contributions of the various processes. This demonstrates that such LCA studies can be used to identify general trends but that specific results should be regarded with respect to the scope and various assumptions made during the assessment. Furthermore, it highlights the importance of transparency about study assumptions as well as applied methods and underlying data to allow for replication and verification of results and adaptation of assumptions to alternative contexts.

### Outlook

For a meaningful interpretation, the number of scenarios had to be restricted. In particular, the panel design presents many possible combinations. The PERC wafers in the silicon submodule could alternatively be a silicon heterojunction (SHJ) or tunnel oxide passivated contact (TOPCon). In fact, SHJ and subsequently TOPCon are projected to displace PERC in the near future.^18^ The assumed improvements in initial power density for all modeled panels are likely in part the result of this shift from PERC to SHJ and TOPcon silicon. For the aim of our study, the inventory for PERC was considered to be sufficiently representative for each of these technologies, though it would be recommended to update the inventory of the silicon submodule once reliable inventories for the industrial production of SHJ and TOPcon become available. Likewise, the perovskite submodule is under continuous development, as novel materials and compositions are tested for improved efficiency and stability. In addition, production, use, and end-of-life could also take place in geographic regions other than assumed herein. Assessing all possible permutations is an exercise beyond the scope of this article. Additional results within the scope of this study can be generated by the reader using the dynamic figures for the process contribution analyses, midpoint to endpoint contribution analyses, and scenario analyses contained in sheets “S2-12” to “S2-14” of Supporting Information 2. The complete LCI model is provided, also enabling the reader to update the model should new data become available or to make any adjustments necessary to generate additional results for scenarios outside the scope of this study. As such, this model could serve as a platform for the future environmental impact assessment of silicon/perovskite tandem photovoltaics. Furthermore, the inventories could be added to premise to enable the assessment of future electricity mixes that account for technological development in the market of photovoltaics beyond simply adjusting technology market shares.

## Data Availability

Additional data sets related to this publication are available from the figshare data repository 10.6084/m9.figshare.c.7233052.

## Supporting Information Available

Supporting Information 1: comprehensive description of the methods and additional results (PDF)
Supporting Information 2: calculations, dynamic LCI model, results, and numerical data for figures in the main text and Supporting Information 1 (XLSX)

## Abbreviations

2T: 2-terminal
4T: 4-terminal
BECCS: bioenergy with carbon capture and storage
BoS: balance of system
DALY: disability-adjusted loss of life years
E: egalitarian
EF: environmental footprint
ENTSO-E: European network of transmission system operators
EVA: ethylene vinyl acetate
GWP100a: global warming potential for a 100 year time horizon
H: hierarchist
I: individualist
IEA PVPS: International Energy Agency Photovoltaic Power Systems Programme
ITO: indium tin oxide
ITRPV: Internal Technology Roadmap for Photovoltaics
LCA: life cycle assessment
LCI: life cycle inventory
LCOE: levelized cost of electricity
PCBM: [6,6]-phenyl C_61_ butyric acid methyl ester
PERC: passivated emitter and rear contact
PET: polyethylene terephthalate
POE: polyolefin elastomer
PV: photovoltaic
PVD: physical vapor deposition
PVT: polyvinyl toluene
RCP: representative concentration pathway
sALD: spatial atomic layer deposition
SDP+TC: slot die printing and thermal cure
SHJ: silicon heterojunction
SnO_2_: tin(IV) oxide
SSP: shared socioeconomic pathway
TOPCon: tunnel oxide passivated contact
