# Supplementary material for: Comparing Environmental Impacts of Single-Junction Silicon and Silicon/Perovskite Tandem Photovoltaics–A Prospective Life Cycle Assessment
Source: ACS Sustain Chem Eng. 2024 May 23;12(23):8860–70. doi: 10.1021/acssuschemeng.4c01952 (PMC11167636; doi:10.1021/acssuschemeng.4c01952)
Supplement: Supplementary file 1 — sc4c01952_si_001.pdf [file sc4c01952_si_001.pdf]

SUPPORTING INFORMATION 1

Comparing environmental impacts of single-junction silicon and silicon/perovskite tandem photovoltaics – a prospective life cycle assessment

Mitchell K. van der Hulst[a,b*], Dorottya Magoss[a], Yiri Massop[a], Sjoerd Veenstra[c], Niels van Loon[c], Ilker Dogan[c], Gianluca Coletti[d,e], Mirjam Theelen[c], Selwyn Hoeks[a], Mark A.J. Huijbregts[a,b], Rosalie van Zelm[a§], Mara Hauck[b,f§]

[a] Department of Environmental Science, Radboud Institute for Biological and Environmental sciences, Radboud University, P.O. Box 9010, Nijmegen, 6500 GL, The Netherlands;
[b] Expertise Group Circularity & Sustainability Impact, TNO, P.O. Box 80015, Utrecht, 3508 TA, The Netherlands;
[c] TNO partner of Solliance, High Tech Campus 21, Eindhoven, 5656 AE, The Netherlands;
[d] School of Photovoltaic and Renewable Energy Engineering, University of New South Wales, Sydney, NSW 2052, Australia;
[e] FuturaSun Holding SRL, Riva del Pasubio 14, Cittadella (PD), 35013, Italy;
[f] Technology, Innovation & Society, Department of Industrial Engineering & Innovation Sciences, Eindhoven University of Technology, P.O. Box 513, Eindhoven, 5600 MB, The Netherlands.

* Email: mitchell.vanderhulst@ru.nl

§ *R.v.Z. and M.H. contributed equally to this paper*

Number of pages: 42

Number of figures: 23

Number of tables: 2

1. DETAILED DESCRIPTION OF THE LIFE CYCLE INVENTORIES

A complete dynamic LCI model is provided in sheet “S2-8. LCIs” of Supporting Information 2. The following sections describe the calculation that underpin this LCI model.

1.1. Production of PERC cell, mono Si M6 wafer & Production of single-junction PERC modules

The LCIs reported in Supplementary Information of Müller et al. [1] were reproduced in this work. The study distinguished between PERC cells and modules produced in China, Germany or Europe. The distinguishing feature is the origin of electricity consumed in each of the unit process, linking the dataset to either electricity from the market group for China (CN), the market for Germany (DE), or the market group for Europe (RER). In addition, production of aluminum alloy and flat glass is regionalized by similarly linking the dataset to electricity production in either of the three regions. In this work, we only consider production of panels in China, since this is where the majority of PV panels are currently produced [2].

The datasets “aluminium alloy production, AlMg3|RER” and “flat glass production, uncoated|RER” were used. New datasets for China were based on these datasets for European production. The new datasets contain an input of European production, a negative input of the electricity consumed in European production and a positive input of electricity of equivalent size from the Chinese electricity market group. Thus, the dataset for aluminum alloy production in China was given an input of 1 kWh from “aluminium alloy production, AlMg3|RER”, an input of −1.59 kWh from “market group for electricity, medium voltage|RER” and an input of 1.59 kWh from “market group for electricity, medium voltage|CN”. Likewise, a dataset for flat glass production in China was given an input of 1 kg from “flat glass production, uncoated|RER”, an input of −0.111 kWh from “market group for electricity, medium voltage|RER” and an input of 0.111 kWh from “market group for electricity, medium voltage|CN”. The confidential front side metallization paste was simply replaced with the dataset for front side metallization paste available in the ecoinvent 3.9.1 database.

The inventory was considered to miss an edge seal. Herein we assumed polyisobutylene is used as edge seal material, for which consumption was calculated based on the dimensions of the panel using Equation S. 1, where PW and PL are the panel width and length respectively, SW is the width of the seal, ST the thickness of the seal and ρ_{PIB} is the density of polyisobutylene, which is 0.88 g/mL [3].

$$Edge\ seal\ per\ panel\ [kg] = (2 * (PW[m] - 2 * SW\ [m]) * SW\ [m] + 2 * PL\ [m] * SW\ [m]) * ST\ [m] * \rho_{PIB}\ [g/ml] * 1000 \left[\frac{kg/m^3}{g/mL}\right] \quad \text{Equation S. 1}$$

Figure S. 1 provides a visual representation of how to identify PW, PL and SW for a panel of 1.6 m^2. The black area is multiplied with an edge seal thickness (ST) of 1.07 mm for the single-junction PERC panel, which is equal to the thickness of the PERC cell and the encapsulant. Butadiene was used as a proxy for polyisobutylene.

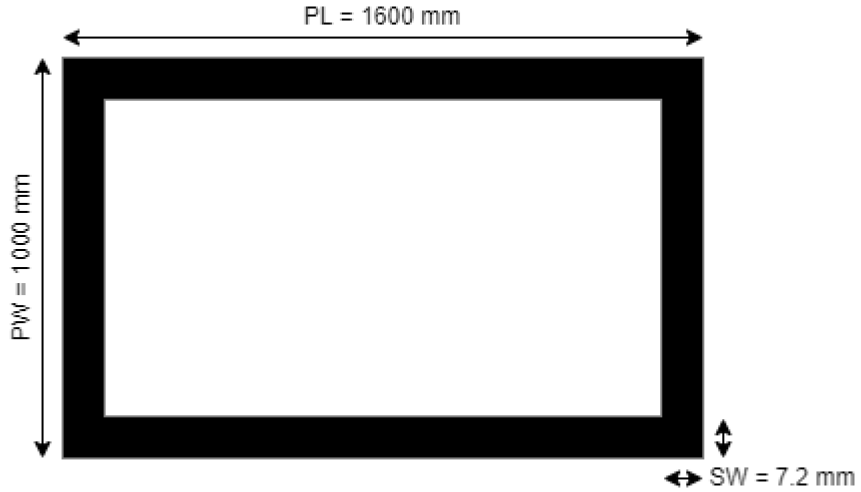

Figure S. 1 Visual representation for calculating the amount of edge seal used in a 1.6m (PL) by 1m (PW) panel with an edge seal width (SW) of 7.2 mm. The black area represents the area of edge seal, which multiplied with the edge seal thickness (ST) gives the total volume of edge seal applied. Note that the seal width is exaggerated and not to scale in this visual representation.

Silicon consumption gets more efficient over time as developments progress to larger ingots and thinner wafers with fewer cutting losses. Silicon consumption was derived from the diameter of the ingot, dimensions of the wafer, thickness of the wafer and thickness of the diamond wire saw. The cross sectional area A_{ingot} of the ingot is calculated from its diameter δ with Equation S. 2.

$$A_{ingot} = \pi * \left(\frac{\delta}{2}\right)^2$$ *Equation S. 2*

Silicon consumption per wafer $m_{Si,wafer}$ is subsequently calculated with Equation S. 3 where x_{wafer} and x_{kerf} are the thickness of the wafer and wire saw, respectively, and δ_{Si} is the density of silicon, which is 2300 kg/m^3.

$$m_{Si,wafer}\ \left[\frac{kg}{wafer}\right] = A_{ingot}\ [m^2] * \left(x_{wafer}\ [m] + x_{kerf}\ [m]\right) * \delta_{Si}\ [\frac{kg}{m^3}]$$ *Equation S. 3*

A wafer is semi-square, with corners cut off to maximize the wafer area per ingot area. The total area of the semi-square wafer A_{wafer} is calculated with Equation S. 4, where x is the length of one side of the square wafer and y is the width of the indentation.

$$A_{wafer}\ [m^2] = x^2\ [m^2] - 0.5 * y^2\ [m^2] * 4$$ *Equation S. 4*

The silicon consumption per wafer area $m_{Si,wafer,area}$ is calculated with Equation S. 5

$$m_{Si,wafer,area}\ \left[\frac{kg}{m^2\ wafer}\right] = \frac{m_{Si,wafer}\ \left[\frac{kg}{wafer}\right]}{A_{wafer}\ [m^2]}$$ *Equation S. 5*

1.2. Production of perovskite materials

1.2.1. Electron transport layer – PCBM

The electron transport layer contains [6,6]-phenyl C61 butyric acid methyl ester (PCBM) for which no representative dataset was available in the ecoinvent 3.9.1 database. The LCI for production of this material was copied from supporting information provided by Tsang et al. [4]. Their datasets were composed through linking with the ecoinvent 2.2 database, which contains different datasets and uses different naming conventions than the ecoinvent 3.9.1 database used herein. Names for inventory items listed by the original source were provided in the comments. Exact names of ecoinvent dataset for inventory items were not provided in the original source. Datasets from ecoinvent were therefore selected that provide a close as possible match to the name of the listed inventory item. A correction was applied to the dataset "methyl 4-benzobutyrate p-tosylhydrazone production|RER" . The original source states that 1.39 kg methanol is consumed and 7.22 kg is regenerated. Looking at the dataset for "methyl 4-benzobutyrate production|RER", the same recovery of 7.22 kg of methanol is stated, while the consumption of methanol in this dataset is 8.03 kg. For the latter dataset, the ratio between consumption and regeneration is 90%. It would seem likely that the same ratio applies to the former dataset and that the amount of regenerated methanol is simply an error in copying of the data. Therefore, the amount of recovered methanol in the dataset "methyl 4-benzobutyrate p-tosylhydrazone production|RER" was decreased to (1.39*0.9=) 1.25 kg.

1.2.2. Absorber – Perovskite

The absorber layer contains perovskite with the composition $Cs_{0.15}FA_{0.85}PbI_{2.94}Br_{0.06}$. The production of this layer requires cesium iodide (CsI), formamidinium iodide (FAI), lead(II) bromide ($PbBr_2$) and lead(II) iodide (PbI_2), for which no representative datasets were available in the ecoinvent 3.9.1 database. The LCIs for production of these materials were copied from supporting information provided by Alberola-Borràs et al. [5]. Names for inventory items listed by the original source are provided in the comments. Exact names of ecoinvent dataset for inventory items were not provided in the original source. Datasets from ecoinvent were therefore selected that provide a close as possible match to the name of the listed inventory item. Some listed emissions to water were not included for lack of corresponding biosphere flows. These emissions are: 196.222 grams of Aluminum oxide (listed 2 times), 925.045 grams of Silicon dioxide, and 303.988 grams of Sodium hydroxide in production of cesium iodide, 545.483 grams of nitric acid and 343.317 grams of Potassium bicarbonate in production of lead(II) bromide and 273.314 grams of nitric acid and 434.257 grams of Potassium bicarbonate in production of lead(II) iodide. The omission of these emissions to water should not influence the results, since there are no characterization factors listed in the applied impact assessment methods for the emission of these substances to any water compartment.

1.2.3. Pollucite

Production of cesium iodide used in the perovskite absorber requires an input of pollucite, which is the ore from which cesium is obtained. No representative datasets was available in the ecoinvent 3.9.1 database for the production of pollucite ore. In line with Khalifa et al. [6], we used the dataset for spodumene production as a proxy, since the mining and processing of this mineral ore was considered comparable to that of pollucite. The dataset for spodumene production in ecoinvent 3.8

was used as a proxy to model production of concentrated pollucite ore in Canada [7]. The original dataset for spodumene in ecoinvent v2 was created by combining datasets from similar process, i.e., iron mining, limestone crushing, and limestone milling [8]. For ecoinvent v3.8, this dataset was updated to represent spodumene mined in Australia [9]. As stated in the general comment for this dataset:

> *"The amount of the exchange "Spodumene, in ground" have been recalculated based on the lithium content in raw and concentrated ores from Stamp et al. (2012), from the mining site of Talison, Australia. The exchanges "Gangue, in ground" and "non-sulfidic tailing, off-site" have been added for the sake of mass balance."*

A similar approach was taken to create a dataset for production of pollucite in Canada. The geographic region of Canada was selected, because 55% (120,000 tonne) of the global world reserve of pollucite (220,000 tonne) is located in Canada [10]. Specifically, pollucite is mined in the Tanco mine at Bernic Lake, Manitoba, Canada, with pollucite having a pre-mining average ore grades of 23.3% Cs_2O [11]. Quantities of resources extracted from the ground and surface water, as well as tailings sent to waste treatment were recalculated for pollucite based on the cesium content of the ore and the cesium content of the pollucite in cesium iodide production.

First the cesium content of the pollucite ore from the Tonca mine is calculated with Equation S. 6 where MW_i are molecular weights of compound i.

$$\frac{0.233\left[\frac{kg\ Cs_2O}{kg\ pollucite}\right]}{MW_{Cs_2O}[g/mol]} * 2\left[\frac{mol\ Cs}{mol\ Cs_2O}\right] * MW_{Cs}[g/mol] = 0.21977\left[\frac{kg\ Cs}{kg\ pollucite}\right] \quad \text{Equation S. 6}$$

To produce 1 kg cesium iodide (i.e. 3.849 moles), we require 1.694725 kg pollucite according to Alberola-Borràs, Vidal and Mora-Seró [5]. Using Equation S. 7, where MW_i are molecular weights of compound i, we can derive that the pollucite going into the sulfuric acid digestion has a cesium content of 0.30 kg Cs/kg pollucite.

$$\frac{\left(\frac{1\ [kg\ CsI] * 1000\ \left[\frac{g}{kg}\right]}{MW_{CsI}\ [g/mol]} * 1\left[\frac{mol\ Cs}{mol\ CsI}\right] * MW_{Cs}\left[\frac{g}{mol}\right] * 0.001\ \left[\frac{kg}{g}\right]\right)}{\left(1.694725\ \left[\frac{kg\ concentrated\ pollucite\ ore}{kg\ CsI}\right] * 1\ [kg\ CsI]\right)} = 0.30184\ \left[\frac{kg\ Cs}{kg\ concentarted\ pollucite\ ore}\right] \quad \text{Equation S. 7}$$

Since the pollucite going into the production of cesium iodide has a higher cesium content than the pollucite ore from the Tonca mine, the ore would need to be processed to increase the cesium content to the required level. Assuming the process has a 95% efficiency, as was assumed for spodumene [7], we found with Equation S. 8 that we need 1.44 kg raw pollucite ore from the Tonca mine to produce 1 kg of concentrated pollucite ore.

$$\frac{\left(\frac{0.30184\left[\frac{kg\ Cs}{kg\ concentrated\ pollucite\ ore}\right]}{0.21977\left[\frac{kg\ Cs}{kg\ raw\ pollucite\ ore}\right]}\right)}{95\%} = 1.4457\left[\frac{kg\ raw\ pollucite\ ore}{kg\ concentrated\ pollucite\ ore}\right]$$

Equation S. 8

The cesium content of pure pollucite, i.e. excluding the gangue, was calculated using the elemental composition of pollucite at the Tonca mine as reported by the Mineralogical Society of America [12]. Based on analyses with an electron microprobe, the average of three analyses corresponds to $(Cs_{0.58}Na_{0.15}Rb_{0.04}Ca_{0.02})_{\Sigma=0.79}Al_{0.86}Si_{2.15}O_6 \cdot nH_2O$. Here, we assumed n to be 1, based on the general composition. The cesium content of pure pollucite was calculated with Equation S. 9, where MW_i is the molecular weight of component i. The molecular weight of pure pollucite at the Tonca mine ($MW_{pollucite,Tonca}$) was calculated with Equation S. 10, where n_i is the molar content of component i.

$$\frac{\left(0.58\left[\frac{mol\ Cs}{mol\ pollcuite}\right] * MW_{Cs}\left[\frac{g}{mol}\right]\right)}{MW_{pollucite,Tonca}\left[\frac{g}{mol}\right]} = 0.27299\left[\frac{kg\ Cs}{kg\ concentrated\ pollucite\ ore}\right]$$

Equation S. 9

$$MW_{pollucite,Tonca}\left[\frac{g}{mol}\right] = \sum_i MW_i\left[\frac{g}{mol}\right] * n_i\ [-] = 282.3632\left[\frac{g}{mol}\right]$$

Equation S. 10

The amount of pure pollucite that needs to be extracted from the ground to generate the required amount of concentrated pollucite was calculated with Equation S. 11, again assuming a recovery efficiency of 95% as was done for spodumene [7].

$$\frac{\left(\frac{0.30184\left[\frac{kg\ Cs}{kg\ concentrated\ pollucite\ ore}\right]}{95\%}\right)}{MW_{pollucite,Tonca}\left[\frac{g}{mol}\right]} = 1.1639\left[\frac{kg\ pure\ pollucite}{kg\ concentrated\ pollucite\ ore}\right]$$

Equation S. 11

Since there are no characterization factors for the use of pollucite, in ground, as a resource, the pollucite was further subdivided into its elemental constituents. The amount of cesium, sodium, rubidium, calcium, aluminum and silicon extracted from the ground were calculated with Equation S. 12.

$$m_i \left[\frac{kg\ i}{kg\ concentrated\ pollucite\ ore}\right] = \left(\frac{1.1639\ \left[\frac{kg\ pure\ pollucite}{kg\ concentrated\ pollucite\ ore}\right]}{MW_{pollucite,Tonca}\ \left[\frac{g}{mol}\right]}\right) * n_i\ \left[\frac{mol}{mol}\right] * MW_i\ \left[\frac{g}{mol}\right] \quad \text{Equation S. 12}$$

The hydrogen and oxygen in the pollucite were added to the gangue. The total amount of gangue was calculated with Equation S. 13.

$$m_{gangue} \left[\frac{kg\ gangue}{kg\ concentrated\ pollucite\ ore}\right] = 1.4457\ \left[\frac{kg\ raw\ pollucite\ ore}{kg\ concentrated\ pollucite\ ore}\right] - \sum_i m_i\ \left[\frac{kg\ i}{kg\ concentrated\ pollucite\ ore}\right] \quad \text{Equation S. 13}$$

The volume of water going in the process of producing concentrated pollucite ore and the mass of non-sulfidic tailing waste coming out of the process were calculated with Equation S. 14 and Equation S. 15 respectively, where a tailing dry mass of 0.7 kg/kg wet tailing was assumed as was done for spodumene [7] and where $m_{PM,i}$ is the mass of emitted particulate matter of size i.

$$m_{water} \left[\frac{m^3\ water\ from\ river}{kg\ concentrated\ pollucite\ ore}\right] = m_{tailing} * (1 - 0.7) * 0.001 \left[\frac{m^3}{kg}\right] \quad \text{Equation S. 14}$$

$$m_{tailing} \left[\frac{kg\ non-sulfidic\ tailing}{kg\ concentrated\ pollucite\ ore}\right] = \left(1.4457\ \left[\frac{kg\ raw\ pollucite\ ore}{kg\ concentrated\ pollucite\ ore}\right] - 1\ [kg\ concentrated\ pollucite\ ore] - \sum_i m_{PM,i} \left[\frac{kg\ PM_i}{kg\ concentrated\ pollucite\ ore}\right]\right) * \left(\frac{1}{0.7}\right) \quad \text{Equation S. 15}$$

The dataset for 1 kg spodumene [7] was used as template for the dataset for 1 kg pollucite. The amount of “spodumene, in ground” was replaced with the amounts of cesium, sodium, rubidium, calcium, aluminum and silicon as calculated with Equation S. 12. The amounts of “gangue in ground”, “water, well, in ground”, and “non-sulfidic tailing, off-site” were replaced with values calculated in Equation S. 13-Equation S. 15. Instead of water from a well, we assumed water from Bernic Lake, MB to be used in the operations and therefore substituted “Water, well, AU” for “Water, lake, CA-MB”. Datasets for energy consumption were changed from Australia to region specific dataset for Canada. The Tanco mine is located in the province of Manitoba, at the border with the province of Ontario. Based on a map of the Canadian electricity grid, electricity was assumed to be generated in Ontario and thus the ecoinvent dataset “Electricity, medium voltage (CA-MB)| market for” was selected. For central and small-scale heat, no dataset was available for the province of Manitoba. Therefore, we used data for Ontario by selecting the dataset “Heat, central or small-scale, other than natural gas (CA-ON)| heat and power co-generation, biogas, gas engine”. Likewise, for district or industrial heat,

the dataset “Heat, district or industrial, other than natural gas (CA-ON)| heat and power co-generation, wood chips, 6667 kW, state-of-the-art 2014” was selected.

1.2.4. Encapsulant

TNO uses a polyolefin elastomer (POE) based encapsulant film in their production of the 4T silicon/perovskite tandem. Herein, we assumed the POE consists of ethylene-octene co-polymer with a 10wt.% octene content and a density of 0.9 g/cm^3. The amount of moles of poly(ethylene-co-octene) per 1 m^2 of encapsulant film is calculated using Equation S. 16 by dividing the mass of 1 m^2 of film by the molecular weight of the poly(ethylene-co-octene). The mass of 1 m^2 of film was calculated by multiplying the density of the encapsulant ($\delta_{encapsulant}$) with the thickness of the film (Δ_{film}) and the required area of 1 m^2. The molecular weight of the encapsulant was derived from the sum of molecular weights of the monomers (MW), multiplied with their respective molar content ($mol\%$).

$$n_{poly(ethylene-co-octene)}\,[mol] = \frac{\delta_{encapsulant}\left[kg/m^3\right] * \Delta_{film}[m] * 1[m^2] * 1000[g/kg]}{(100\% - 10mol\%) * MW_{ethylene}[g/mol] + 10\ mol\% * MW_{1-octene}[g/mol]} \qquad \text{Equation S. 16}$$

This molar mass is subsequently used to derive the quantities of ethylene and 1-octene required for 1 m^2 of encapsulant film using Equation S. 17 and Equation S. 18 and the molecular weights of the monomers (MW).

$$Ethylene\ [kg] = \frac{(100\ \% - 10\ mol\%) * n_{poly(ethylene-co-octene)} * MW_{ethylene}[g/mol]}{0.001\ [kg/g]} \qquad \text{Equation S. 17}$$

$$Octene\ [kg] = \frac{10\ mol\% * n_{poly(ethylene-co-octene)} * MW_{octene}[g/mol]}{0.001\ [kg/g]} \qquad \text{Equation S. 18}$$

The ecoinvent dataset “market for n-olefins|GLO” was used to represent inputs and outputs from production of the 1-octene. An extrusion process was included to account for production of the encapsulant film.

1.2.5. Current collector tape

For conduction of charge from the side of the module to the electrodes, two pieces of solar tape are used, each 4 cm wide and 1.2 long. Assumed dimensions were obtained from technology experts at TNO. The solar tape was modelled after the 3M™ Charge-Collection Solar Tape 3007 using an available datasheet for this product [13]. This solar tape consists of a 0.023 mm thick conductive acrylic adhesive on a 0.035 mm thick tin plated copper backing and comes with a 0.050 mm thick 1-side Si-coated PET release liner. The thickness of the tin plating was assumed to be 0.005 mm, fully encasing the copper. For these data, a unit process was constructed for 1 m of current collector tape. The amount of adhesive required per 1 meter of tape was calculated using Equation S. 19, with $\Delta_{adhesive}$ and $\delta_{adhesive}$ being the thickness and density of the adhesive layer, respectively. For the density, the experimental/literature value for poly(methyl methacrylate) (PMMA) was used as an approximation for the density of the adhesive [14].

$$Adhesive\ [kg] = \Delta_{adhesive}[m] * width_{tape}[m] * 1\ [m] * \delta_{adhesive}[g/mL] * 1000\left[\frac{kg/m^3}{g/mL}\right] \qquad \text{Equation S. 19}$$

The conductive adhesive is an acrylic pressure sensitive adhesive (PSA) with undisclosed composition. A typical acrylic PSA consists of different monomers in varying compositions: 70-90% soft monomer such as 2-ethyl hexyl acrylate, n-butyl acrylate, or iso-octyl acrylate; 0-30% hard monomer such as methyl methacrylate, methyl acrylate, vinyl acetate, or styrene; 3-10% polar monomer such as acrylic acid, 2-hydroyl ethyl acrylate, or n-vinyl pyrrolidone [15]. From the listed soft co-monomers, an ecoinvent dataset is only available for n-butyl acetate, while for the polar co-monomer, a dataset is only available for acrylic acid. For simplicity, we assumed the acrylic PSA to be 100% n-butyl acrylate. This assumption was considered justifiable, since the hard monomers methyl methacrylate and methyl acrylate have endpoint footprints around 50% larger than those of n-butyl acetate, while the polar monomer acrylic acid has endpoint footprints around 50% smaller than those of n-butyl acetate. Therefore, an acrylic PSA with an 80:10:10 content of soft:hard:polar monomer would have endpoint footprints roughly equal to that of the soft co-polymer.

For the backing, the width ($width_{copper}$) and thickness ($\Delta_{copper}[kg]$) of the copper were calculated using Equation S. 20 and Equation S. 21, accounting for the tin plating.

$$width_{copper}\ [m] = \ width_{tape}[m] - 2 * \ \Delta_{plating}[m] \qquad \text{Equation S. 20}$$

$$\Delta_{copper}[kg] = \ \Delta_{backing}[m] - 2 * \Delta_{plating}[m] \qquad \text{Equation S. 21}$$

Therefore, the amount of copper required per 1 meter of tape was calculated using Equation S. 22, with δ_{copper} being the density of copper.

$$Copper\ [kg] = \ \Delta_{copper}[m] * width_{copper}[m] * 1\ [m] * \delta_{copper}[g/cm^3] * 1000 \left[\frac{kg/m^3}{g/cm^3}\right] \qquad \text{Equation S. 22}$$

The amount of tin required is found with Equation S. 23 by subtracting the volume of copper from the volume of the backing and multiplying with the density of tin (δ_{tin}).

$$Tin\ [kg] = (\Delta_{backing}[m] * width_{tape}[m] * 1\ [m]\ -\ \Delta_{copper}[m] * width_{copper}[m] * 1\ [m]) * \delta_{tin}[g/cm^3] * 1000 \left[\frac{kg/m^3}{g/cm^3}\right] \qquad \text{Equation S. 23}$$

For the release liner, the amount of silicon was calculated using Equation S. 24 and a whitepaper on silicon release coatings from Dow [16], stating that *"Plastic films, with their perfect hold-out and smooth surface, need only about 0.1 to 0.2 grams per square meter (gsm) to effect perfect film coverage and exhibit good release properties"*. Here, we have taken an average coating of 0.15 gsm.

$$Silicon\ [kg] = 0.15\ [g/m^2] * 0.001\ [kg/g] * width_{tape}[m] * 1\ [m] \qquad \text{Equation S. 24}$$

The ecoinvent dataset “market for silicone product|RER” was used to represent inputs and outputs from production of the silicon used in the coating. Processing requirements for the coating process itself were omitted for lack of data.

The amount of PET required was derived with Equation S. 25 by subtracting the volume of silicon coating from the volume of the release liner and multiplying with the density of PET (δ_{PET}). The experimental/literature value for the density of poly(ethylene terephthalate) (PET) was obtained from polymerdatabase.com [17]. For the volume of the silicon coating, again the Dow white paper was used [16], which states that *"a coating of 1 gsm is almost exactly 1 micron (one millionth of a meter) thick"*.

$$PET\ [kg] = (\Delta_{release\ liner}[m] * width_{tape}[m] * 1\ [m]\ -\ 10^{-6} \left[\frac{m}{\frac{g}{m^2}}\right] * 0.15\ [g/m^2] * width_{tape}[m] * 1\ [m]) * \delta_{PET}[g/mL] * 1000 \left[\frac{kg/m^3}{g/mL}\right] \quad \text{Equation S. 25}$$

For the release liner, an extrusion process was included to account for production of the PET film and municipal incineration of mixed plastic waste was included to account for end-of-life waste treatment of the film. The latter is a conservative assumption, since waste release liner could also be collected for mechanical or chemical recycling.

1.2.6. Confidential layers

Confidential information regarding the TNO production process was protected by converting the unit processes for the confidential layers to aggregated LCIs, also referred to as system processes. A unit process describes a distinct part of the life-cycle of a product, such as the production of a distinct material. Direct inputs and outputs from both the biosphere and other unit processes in the technosphere are included in such datasets. This makes it possible to track environmental impacts throughout the foreground (product system) and background (wider economy) system. While such traceability is generally preferred, it could result in disclosure of confidential information, e.g., the use of a specific material that provide a competitive edge to a producer. For a system process, all related unit processes in the foreground and background are assessed and their environmental flows (i.e. flows to and from the biosphere) are aggregated. A system process therefore only contains biosphere flows, making it impossible to trace back the origin of impacts to specific unit processes in the technosphere. This system dataset will give the exact same result as the corresponding unit process in life cycle impact assessment.

Unit datasets for the materials used in hole transport layer and interface layer were created for internal use. These materials were both ink based for application with slot die coating. Consumption of ink per square meter was calculated using data from the cost model CostInsight developed at TNO for internal use. The LCIs for the constituents of the inks were derived from literature sources that described the synthesis processes for these constituents. Representative datasets from the ecoinvent database were selected wherever possible. For substances or processes with no representative dataset in ecoinvent, process trees were constructed to investigate their production further upstream. The end of each branch in this process tree consisted solely of substances and processes present in either the ecoinvent 3.9.1 or biosphere3 databases. When literature provided no data on energy consumption in the production processes of the used substances, energy consumptions were estimated based on the average electricity and heat consumption for a wide range of chemicals at the Gendorf Chemical plant as proposed by Hischier et al. [18]:

"Based on the information in the Environmental Report (Gendorf 2000) of a chemical plant site of several chemical production companies in Germany (Werk GENDORF – 12 companies, producing 1,500 different products), average consumption of electricity and heat are calculated and used as default values."

The latest figures for the Gendorf chemical plant site were used to estimate energy consumption for present day chemical production [19]. In 2020, the plant was reported to consume 4.554.990 GJ natural gas and 3.693.900 GJ electricity to produce 1.511.100 tonnes of products. This translates to an energy consumption of 3.01 MJ natural gas/kg chemical product and 0.679 kWh electricity/kg chemical product.

To share the dataset for the confidential layers, their unit process datasets were converted to a cumulative LCI dataset to hide the sensitive data contained in the unit processes. First, unit process datasets were created in Activity Browser for materials consumed in both layers, thus excluding energy inputs from the slot die coating and thermal anneal processes. A scenario LCA was conducted using the scenario difference file from premise to obtain results for production of these confidential materials in 2023 to 2050. The list of biosphere flows under the “Inventory” tab of the “LCA results” tab in Activity Browser were exported to excel. The LCI list was subsequently transferred to an empty database template file for Activity Browser. The categories field was processed from the LCI results output format to the database file input format (i.e. *('compartment', 'sub-compartment')* was converted to *compartment::sub-compartment*). The database file was locally stored and subsequently imported in activity browser as a new database. Finally, the LCI dataset was copied to the sheet *S2-4. LCI* of Supporting Information 2.

1.3. Production of perovskite sub-module

The perovskite solar cell (PSC) as presented in Figure 1 of the main text was created by sequential deposition of each layer on top of the substrate low-iron solar glass. The hole transport layer is deposited on this substrate before the electron transfer layer, giving the perovskite a positive-intrinsic-negative (PIN) configuration. This configuration is preferred over negative-intrinsic-positive (NIP), since atomic layer deposition of the electron transport layer provides good coverage and mechanical and thermal stability to the perovskite layer. This enables the sputtering of the ITO back contact layer, which is required in tandem applications for its transparency. Cells are formed and monolithically series interconnected by placing three scribes through laser ablation after deposition of the top contact and before and after deposition of the bottom contact.

The cost model CostInsight developed at TNO for internal use was used to derive material and energy consumptions for the production of 1 m^2 of perovskite sub-module. A detailed account for calculated consumptions for both the pilot scale and industrial scale production of the perovskite-sub-module is produced in sheet *S2-2. Calculations – TNO* of Supporting Information 2. The sub-module is created layer by layer, with each step represented by its own unit process. Process parameters present in CostInsight were obtained from industry, either from public data sources or through personal communication. Since the perovskite sub-module is produced in sequential steps in a sheet-to-sheet fashion, it was assumed that defects could be removed directly after the process that resulted in its defect. In other words, if a defect arises at processing step 4, the sub-module is removed, thus wasting only materials and energy from steps 1 to 4 while avoiding further wasting materials and energy in subsequent processing steps.

The mass m per square meter for each layer i in the perovskite sub-module was calculated using Equation S. 26, where $height_i$ is the thickness and δ_i the density of layer i.

$$m_{layer,i}\ [kg/m^2] = height_i\ [m] * \delta_i\ [kg/m^3] \qquad \textit{Equation S. 26}$$

For the absorber layer, four precursor materials are mixed to obtain cesium formamidinium lead halide perovskite. The absorber modelled in this work has a composition of $Cs_{0.15}FA_{0.85}PbI_{2.94}Br_{0.06}$, meaning there are 15% cesium ions and 85% formamidinium ions in the A position, 100% lead in the B position and 98% iodide and 2% bromide in the X position of the ABX_3 crystal lattice. The molecular weight of one unit cell of the cesium formamidinium lead halide perovskite crystal lattice was calculated with Equation S. 27, where MW_i is the molecular weight of each constituent and n_i is its occurrence in the crystal lattice.

$$MW_{perovskite}\ [g/mol] = \sum_i MW_i * n_i \qquad \textit{Equation S. 27}$$

The mass of each precursor required to satisfy the demand of $m_{layer,perovskite}$ is calculated using Equation S. 28, where MW_i is the molecular weight of precursor i and ME_i describes the amount of moles of precursor i that is required to produce 1 mol of perovskite.

$$m_{precursor,i}\ [kg] = \frac{m_{layer,perovskite}\ [kg]}{MW_{perovskite}[\frac{kg}{mol}]} * ME_i \left[\frac{mol_i}{mol_{perovskite}}\right] * MW_i[\frac{kg}{mol}] \qquad \textit{Equation S. 28}$$

Cesium iodide (CsI) is the only source of cesium for perovskite, so its molar equivalent consumption is determined by the amount of cesium ions in the crystal lattice of perovskite, i.e. 0.15 mol CsI/mol perovskite. Likewise, the amount of formamidinium iodide (FAI) required is 0.85 mol FAI/mol perovskite. Lead(II) bromide ($PbBr_2$) contains two bromide ions per molecule and is the only source of bromide, so the amount required is (0.06/2=) 0.03 mol $PbBr_2$/mol perovskite. Lead(II) iodide (PbI_2) contains two iodide ions per molecule. The amount of iodide required is equal to the molar content of iodide in the Perovskite, minus the iodide already supplied by cesium iodide and formamidinium iodide. Therefore, the amount of lead(II) iodide required is ((2.94−0.15−0.85)/2=) 0.97 mol PbI_2/mol perovskite.

For the slot die coating processes, the mass of consumed solvent $m_{solvent,i}$ was calculated using Equation S. 29, where WDR_i is the ratio between the mass of the solvent (wet) and solutes (dry) in the ink (Equation S. 30).

$$m_{solvent,i}\ [kg] = m_{layer,i}\ [kg] * (WDR_i - 1) \qquad \textit{Equation S. 29}$$

$$WDR_i = \frac{m_{ink\ solvent}\ [m]}{m_{ink\ solutes}\ [m]} \qquad \textit{Equation S. 30}$$

For sputtering processes, the sputtering target will not fully be utilized. Furthermore, not all sputtered material will deposit as a layer on the perovskite submodule. Likewise, for slot die coating process, some of the ink will be wasted during the deposition process. These losses were accounted for using Equation S. 31, where the mass of consumed materials $m_{consumed,i}$ is calculated by dividing

the mass of the layer or solvent $m_{layer\ or\ solvent,i}$ by the target utilization η_{target} and deposition efficiency $\eta_{deposition}$. Consequently, the mass of the waste materials ($m_{waste\ material,i}$) or waste solvents ($m_{solvent,i}$) is given by Equation S. 32

$$m_{consumed,i}\ [kg] = \frac{m_{layer\ or\ solvent,i}\ [kg]}{\eta_{target}\ [\%] * \eta_{deposition}[\%]}$$ *Equation S. 31*

$$m_{waste\ material/solvent,i}\ [kg] = m_{consumed,i}\ [kg] - m_{layer/solvent,i}\ [kg]$$ *Equation S. 32*

Processing requirements such as electricity, cooling water, and inert gases were calculated from machine specifications. Available data were obtained for machines that could handle substrates with a width of 1.2, 1.5 or 2 meter depending on the machine. Processing requirements were scaled down to correspond with machinery that could handle substrates with a width of 0.12 m. The equipment $throughput_i$ of equipment i was calculated from the $width_i$ and $speed_i$ using Equation S. 33, which was subsequently scaled down linearly using Equation S. 34.

$$throughput_i\ [m^2/h] = width_i\ [m] * speed_i\ [m/min] * 60\ [min/h]$$ *Equation S. 33*

$$throughput_{scaled,i}\ [m^2/h] = throughput_i\ [m^2/h] * \frac{0.12\ [m]}{width_i\ [m]}$$ *Equation S. 34*

The power consumption listed in the machine specifications was assumed to be the nameplate capacity ($P_{nameplate}$) of the equipment, with optimal power consumption assumed to be 75% of this nameplate capacity [20]. The optimal power consumption was assumed to scale with the width of the substrate $width_i$ according to a power law, with a size scaling factor of 0.7 [21]. Therefore, Equation S. 35 gives the scaled down electricity consumption by equipment i.

$$E_{scaled,i}\ [kWh] = \frac{P_{nameplate}[kW] * 75\% * \left(\frac{0.12\ [m]}{width_i\ [m]}\right)^{0.7}}{throughput_{scaled,i}\ [m^2/h]}$$ *Equation S. 35*

Likewise, consumption of cooling water ($water_i$) is scaled down using a power law (Equation S. 36).

$$water_{scaled,i}\ [m^3] = \frac{water_i\ [m^3/h] * \left(\frac{0.12\ [m]}{width_i\ [m]}\right)^{0.7}}{throughput_{scaled,i}\ [m^2/h]}$$ *Equation S. 36*

And finally, consumption of gas j was scaled down using the power law and the density δ_i of the gas (Equation S. 37), with j representing the gasses argon, oxygen and nitrogen

$$gasses_{scaled,i,j}\ [kg] = \frac{\dfrac{gasses_{i,j}\ \left[\frac{m^3}{yr}\right]}{24\ \left[\frac{h}{day}\right] * 365\ \left[\frac{day}{yr}\right]} * \delta_j\ \left[\frac{kg}{m^3}\right] * \left(\frac{0.12\ [m]}{width_i\ [m]}\right)^{0.7}}{throughput_{scaled,i}\ [m^2/h]}$$ *Equation S. 37*

For the industrial production of the perovskite sub-module, Equation S. 34 to Equation S. 37 were reused, replacing 0.12 m with 1.6 m.

1.4. Production of PERC/perovskite tandem modules

The LCIs of the single-junction PERC modules was used as a starting point for modelling the PERC/perovskite tandems modules. The input of ethylvinylacetate foil as encapsulant was replaced with an input of polyolefin elastomer encapsulant film. Edge seal waste treatment for cardboard box, EUR-pallet, and LDPE packaging film were added while waste treatment for mixed plastic waste and polyvinyl fluoride were removed, as was also done for the dataset of production of single-junction PERC modules. Waste treatment for the module itself was modelled in a separate dataset to account for the time lag between production and end-of-life. Additional inputs of current collector tape and perovskite-sub module were added. The mass of the front glass was deducted from the inventory, since this was already included in the dataset for the perovskite sub-module.

1.5. Production of Balance of System (BoS)

The LCI for the electric installation, inverter and mounting system for slanted- or flat-roof were obtained from [22]. Waste treatment for packaging was included, while waste treatment for the mounting system itself was excluded and instead modelled in a separate dataset to account for the time lag between production and end-of-life.

1.6. Use of single-junction PERC module & Use of PERC/perovskite tandem modules

Transportation of finished panels from the factory to the site of installation was modelled based on dataset provided in the supporting Information of [1]. Transportation datasets have the unit of tonkilometer and thus depend on the transportation distance and the weight of the transported goods. Masses of packaged panels were calculated individually and transportation requirements were adjusted accordingly.

Power densities for the monofacial single-junction panel in 2023-2033 were obtained from Fig. 55 in ITRPV [23] and trends were extrapolated to 2050. The final power density is 24% which is well under the Shockley-Queisser limit of 33.7% and was therefore considered feasible. The power density for the pilot scale monofacial tandem device was obtained from Coletti et al. [24], while projections for the power densities at the industrial scale in 2027-2033 were obtained from ITRPV [23]. Again, trends were extrapolated to 2050 and the final power density of 30.7% is well under the theoretical upper limit of 45%, thereby being considered feasible. For the bifacial devices, increased power densities of +17.5% and +15% were assumed for the single-junction and tandem panels respectively. These increases were derived from Table 1 in [24], where the power density of the single-junction device increases from 22.8% to 26.8% when going from 100mW/cm^2 front-incident irradiance to 100mW/cm^2 front- and 20 mW/cm^2 rear-incident irradiance and the where the power density of the 4T tandem device increases from 26.5% to 30.5% when going from 100mW/cm^2 front-incident irradiance to 100mW/cm^2 front- and 20 mW/cm^2 rear-incident irradiance. It was assumed the monofacial to bifacial power density ratio would remain the same over time.

1.7. End-of-life of Balance of System (BoS)

New datasets were created for waste treatment of the electric installation, inverter and mounting system for slanted- or flat-roof. Each material contained in these balance-of-system components was

assumed to become waste at end-of-life and to be processed by the respective markets for these wastes. For example, the electric installation contains 20 grams of copper, thus an input of 20 grams from the market for copper scrap was included in the dataset for waste treatment of the electrical installation at end of life. These market datasets contain contributions for waste transport and, in the case of final disposal, they also contain contributions related to the method of final disposal (e.g. carbon dioxide emissions from plastic incineration). However for recyclable materials, the recycling itself, as well as avoided burdens from recovered materials are excluded, in line with the cut-off modelling approach. This results in low contributions for metal waste streams, which are generally recycled. The end-of-life processes for the balance-of-system do not include contributions from the disassembly in its constituent materials for lack of data, thus underestimating contributions from the processing of balance-of-system components at end-of-life.

1.8. End-of-life of single-junction PERC modules & End-of-life of PERC/perovskite tandem modules

Material and energy inputs and waste and emission outputs of the FRELP recycling scheme were derived from Figure 1 in Latunussa et al. [25]. The unit process of the recycling scheme is defined on a per-kilogram basis. Additional datasets were created to convert 1 kilogram to 1 square meters, to 1 kilowatt peak, to a 3kWp installation, and finally to 1 kWh of electricity produced so that the impacts from recycling can easily be added to the impacts from production. Quantities were adjusted to align with the bill of material of the studied panels. Calculation are provided in Supporting Information 2.

2. VISUALIZATION OF LIFETIME ELECTRICITY OUTPUT FOR HIGH ANNUAL DEGRADATION RATES

Figure S. 2 displays annual electricity yield from monofacial panels produced in 2050 over their 40-year lifetime. The black line represents the electricity output of a monofacial single-junction silicon panel, which shows a gradual decline as a result of the assumed 0.4% (relative) annual degradation rate. The blue line represents the electricity output for a monofacial silicon/perovskite tandem panel under the same assumptions. The blue line never drops below the black line and therefore no refurbishment is included in this scenario. Increasing the annual degradation rate to 1% (relative) is represented by the green line. This line has a steeper slope and therefore the green line would drop below the black line in 2081, as represented by the dashed line. Through refurbishment, the annual electricity output is restored to its original value, thus the annual electricity output of the tandem panel does not drop below that of the single-junction device. The yellow line represents an annual degradation rate of 2% (relative) and therefore has an even steeper slope. Its annual electricity output would drop below that of the single-junction device in 2062, as represented by the dashed line. Again, the panel is refurbished to increase the annual electricity output to its initial value. However, due to the high annual degradation rate, the yellow line would drop below the black line again in 2079, requiring a second round of refurbishing. Finally, the red line represents an annual degradation rate of 3% (relative), requiring three rounds of refurbishing to ensure that the annual electricity output stay above that of the single-junction alternative. The lifetime electricity output is simply the area under the curve for each of the five scenarios. Refurbishment thus ensures that the lifetime electricity output for the tandem device is always higher than that of the single-junction device.

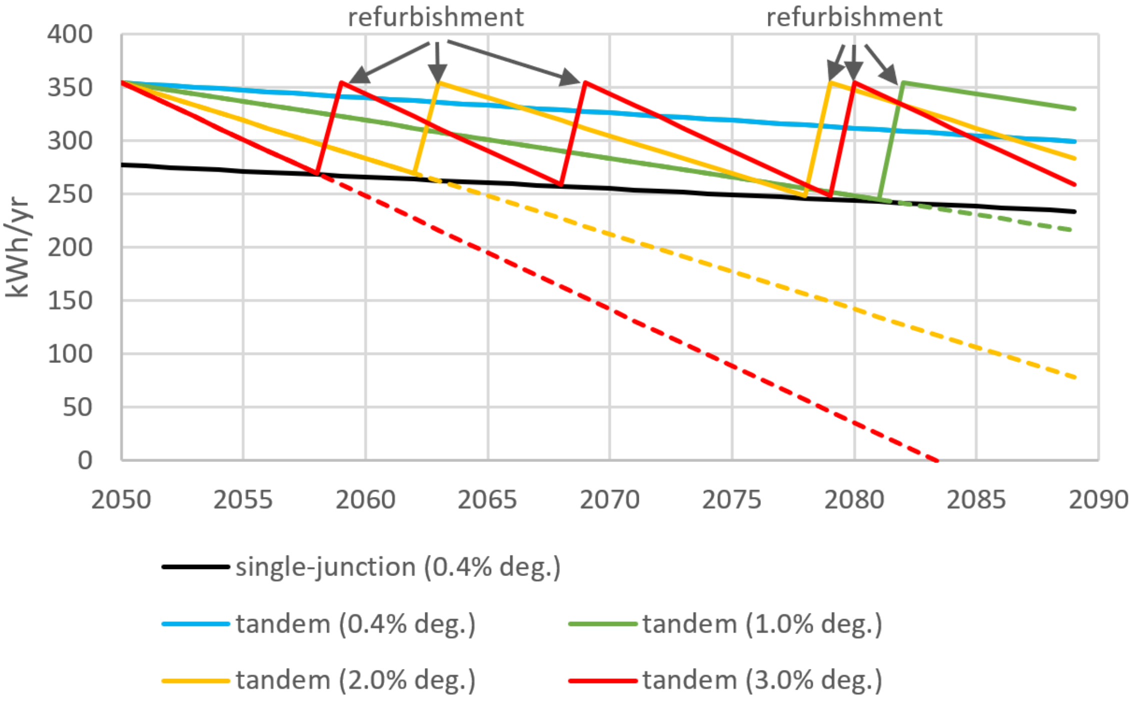

Figure S. 2 *Annual electricity output for a monofacial panel produced in 2050 under various assumptions for the degradation rate of the perovskite in the monofacial tandem panel.*

3. DETAILED DESCRIPTION OF THE PROSPECTIVE ASSESSMENT

Table S. 1.*Overview of application of the prospective LCA framework in van der Hulst, Huijbregts, van Loon, Theelen, Kootstra, Bergesen and Hauck [21]. TRL: technology readiness level see for definitions [26, 27]; MRL: manufacturing readiness level see for definitions [27]; MPL: market penetration level; PERC: passivated emitter and rear contact; SHJ: silicon heterojunction; TOPCon: tunnel oxide passivated contact; WEEE: waste electrical and electronic equipment.*

Phase: Step	System	Current state	Future state
**Phase I:**<br>Definition of development stage	• Production of monofacial and bifacial silicon modules	• Industrial scale production: TRL 9; MRL 10; MPL 80%.<br>*MPL for PERC in 2022 [23], while PV has an MPL of 43% in the renewable energy market [28].*	• Industrial scale production: TRL 9; MRL 10; 0%.<br>*Silicon PV market share of PERC in 2033 is ~10% with SHJ and TOPCon eventually pushing it out the market [23].*
	• Production of monofacial and bifacial silicon/perovskite modules	• Monofacial: pilot line at full scale in a relevant operational environment [29]: TRL 7; MRL7; MPL 0%<br>• Bifacial: prototype level in a simulated environment (internal data from TNO): TRL 5; MRL 5; MPL 0%	• Industrial scale production:TRL 9; MRL 10; MPL >5%.<br>*Si-based tandems enter the market after 2025 and have a market share of ~5% in 2033 [30]. Overall MPL of bifacial modules in 2033 is 70% [30].*
	• Balance of system (BoS)	• Industrial scale production: TRL 9; MRL 10; MPL 100%.<br>*No distinction is made in specific technologies.*	• Industrial scale production: TRL 9; MRL 10; MPL 100%.<br>*No distinction is made in specific technologies.*
	• End-of-life waste recycling for all four product systems (excl. silicon and silver recovery)	• Industrial scale production: TRL 9; MRL 10; MPL 100%.<br>*MPL is assuming all European panels are recycled, in line with the European directive on WEEE.*	• Industrial scale production: TRL 9; MRL 10; MPL 0%.<br>*Assuming recycling with silicon and silver recovery becomes dominant.*
	• End-of-life waste recycling for monofacial and bifacial silicon modules (incl. silicon and silver recovery)	• Final stages have been studied at the laboratory scale and are currently studied for industrialization [31]:<br>TRL 5; MRL 5; MPL 0%.	• Industrial scale production: TRL 9; MRL 10; MPL 100%.<br>*Assuming recycling with silicon and silver recovery becomes dominant.*
	• End-of-life waste recycling for monofacial and bifacial silicon/perovskite modules (incl. silicon and silver recovery)	• Proof of concept at laboratory scale [32]:<br>TRL 3; MRL 3; MPL 0%.	• Industrial scale production: TRL 9; MRL 10; MPL 100%.<br>*Assuming recycling with silicon and silver recovery becomes dominant.*
**Phase: Step**	**Modelled change**	**Current state**	**Future state**
**Phase II:**<br>Process changes	• Improved coverage and mechanical and thermal stability<br>• Utilisation of safer solvent [33, 34]	• Slot die printing of the tin(IV)-oxide (SnO_2) electron transport layer (ETL)<br>• Dimethylformamide (DMF) in production (slot die printing of the absorber) and end-of-life (dissolution of the absorber)	• Spatial atomic layer deposition (sALD) of the tin(IV)-oxide (SnO_2) electron transport layer (ETL)<br>• Dimethyl sulfoxide (DMSO) in production (slot die printing of the absorber) and end-of-life (dissolution of the absorber)
**Phase II:**<br>Size scaling	Product scaling in silicon/perovskite tandem production	Pilot scale product: 12 cm x 12 cm (i.e. 0.0144 m^2)	Industrial scale product: 100 cm x 160 cm (i.e. 1.6m^2)
**Phase II:**<br>Process synergies	Recovery and re-use of materials from waste solar panels	Recovery of glass, aluminium, and copper [1, 35]	Recovery of glass, aluminium, copper, silicon, and silver [25]
**Phase III:**<br>Industrial learning	Increased lifetime electricity output	The International Technology Roadmap for Photovoltaics [30] was consulted for projections over the period 2023-2033 on developments in the main contributors to the footprint, which are power density, degradation rate, lifetime, cell area, and thickness of silicon wafer. For the years 2035, 2040, 2045, and 2050, degradation rates, lifetime, and cell areas were kept constant at 2033 levels, while power density and thickness of silicon wafer were extrapolated based on trends.	
**Phase III:**<br>External developments	Decarbonization of electricity generation, truck transport, and steel, cement, and fuel production	The LCI database ecoinvent 3.9.1 [36] was converted to a superstructure database with scenario differences file [37] using the python software *premise* [38]. Scenarios were derived from Integrated Assessment Model *IMAGE* [39] for Shared Socioeconomic Pathway 2 *“Middle of the road”* [40], assessing the baseline scenario, as well as those described by Representative Concentration Pathways 2.6 and 1.9 [41]. Scenarios for production were created for years 2023, 2027, 2030, 2033, 2035, 2040, 2045, and 2050, while scenarios for end-of-life waste recycling were created for years 2053, 2060, 2070, 2073, 2075, 2080, 2085, and 2090 to account for the 30 to 40 year time lag between production and end-of-life waste treatment [42].	

4. ADJUSTMENTS TO THE CLIMATE CHANGE IMPACT ASSESSMENT METHOD

Throughout this work, we have used an adapted version of the ecoinvent database which was generated using premise [38]. The premise tool makes several adjustments to existing datasets in the ecoinvent database as well as introduces several new datasets, depending on which integrated assessment model (IAM) and scenarios were selected in the transformation process. Some of these scenarios rely on bioenergy with carbon capture and storage (BECCS), direct air capture (DAC) and other forms of storage that use biogenic or atmospheric carbon resources. The respective datasets contain some elementary flows relevant to the midpoint impact category of climate change for which no characterization factors are defined in the default life cycle impact assessment methods available in brightway2 and the Activity Browser.

Absence of these characterization factors would result in these elementary flows simply not being considered, resulting in an over- or underestimations of the contribution to climate change of some processes. The following characterization factors for carbon dioxide should be added:

"Carbon dioxide, in air", with a characterization factor of −1.

"Carbon dioxide, non-fossil, resource correction", with a characterization factor of −1.

"Carbon dioxide, non-fossil", with a characterization factor of 1.

In addition, a correction is made to the characterization factor of biogenic methane. In the default IPCC impact assessment methods its characterization factor is equal to that of fossil methane, minus 2 kg CO_2-eq, in line with the IPCC 2013 method as presented in Myhre et al. [43]. However, as Muñoz and Schmidt [44] have demonstrated, this difference in characterization factors should actually be 2.75 kg CO_2-eq. independent of the time horizon of the impact assessment method. The characterization factors for biogenic methane have therefore been adjusted accordingly in the ReCiPe 2016 method, while the set of LICA methods from the IEA PVPS already include this 2.75 kg CO_2-eq. difference between fossil and biogenic methane.

Some of the IAM scenarios introduce technologies that require a considerable consumption of hydrogen, which leads to significant emissions of hydrogen due to process losses. While hydrogen itself is not a greenhouse gas, its chemical reactions change the abundance of greenhouse gases methane, ozone, and stratospheric water, as well as aerosols [45]. A characterization factor of 37.3 ±15.1, 11.6 ±2.8 , and 3.31 ± 0.98 kg CO_2-eq. was modelled by Sand et al. [45] for the global warming potential over a 20 (GWP20), 100 (GWP100), and 500 (GWP500) year time horizon, respectively.

Throughout this work, the life cycle impact assessment methods ReCiPe 2016 and the set of methods recommended by the IEA PVPS were used. The adjustments described above were made to the midpoint impact category of climate change for each of the three narratives (i.e. individualist, hierarchist, or egalitarian). For the egalitarian narrative, a time horizon of 1000 years is assumed in the ReCiPe 2016 life cycle impact assessment method [46]. For simplicity, it was assumed that the GWP500 for hydrogen is equal to its GWP1000, which is likely to result in an overestimation of the characterization factor in the egalitarian narrative as the GWP becomes lower for longer time horizons. The characterization factors were converted from midpoint to endpoint using the conversion factors in Table 2.3 of Huijbregts et al. [46]. Activity Browser 2.9.6 [47, 48] was used to adapt the characterization factors for the EF v3.1 and the ReCiPe 2016 midpoint and endpoint life cycle impact assessment methods. An overview of adjustments to the midpoint and endpoint categories is provided in Supporting Information 2, sheet "S2-9. CC changes CFs".

5. ADDITIONAL RESULT FIGURES

5.1. Midpoint-to-endpoint contribution analysis – monofacial panel – ReCiPe 2016 Hierarchist (H) – Resource Scarcity

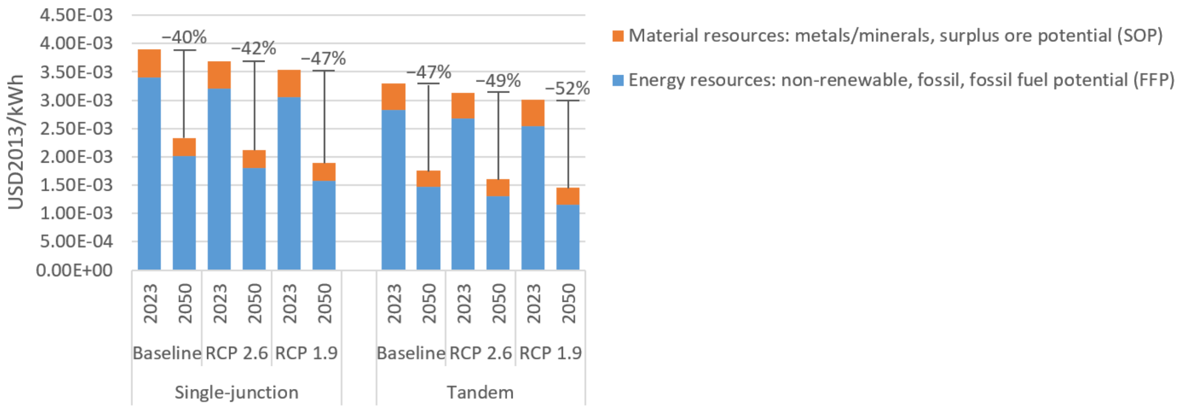

Figure S. 3. *Midpoint-to-endpoint contribution analysis for the endpoints of Resource Scarcity in USD2013/kWh of the hierarchist (H) perspective of the ReCiPe 2016 life cycle impact assessment method. The percentages represent the decrease in impact between 2023 and 2050 for each respective background scenario. These background scenarios were based on Shared Socio-economic Pathway 2 (SSP2), i.e. the "Middle-of-the-Road" development scenario with social, economic and technological developments following historic trends. From this scenario, the baseline scenario and the Representative Concentration Pathways (RCPs) 2.6 and 1.9 were assessed, which correspond with ~3.5°C, 1.6–1.8°C and 1.2–1.4°C global mean surface temperature increase by 2100, respectively. Other assumptions: insolation: 1391 kWh/m²/yr; geographic scope: China (production) and Europe (use and end-of-life); panel design: monofacial; annual degradation rate for perovskite: 0.5% (relative).*

5.2. Midpoint-to-endpoint contribution analysis – bifacial panel – ReCiPe 2016 Hierarchist (H)

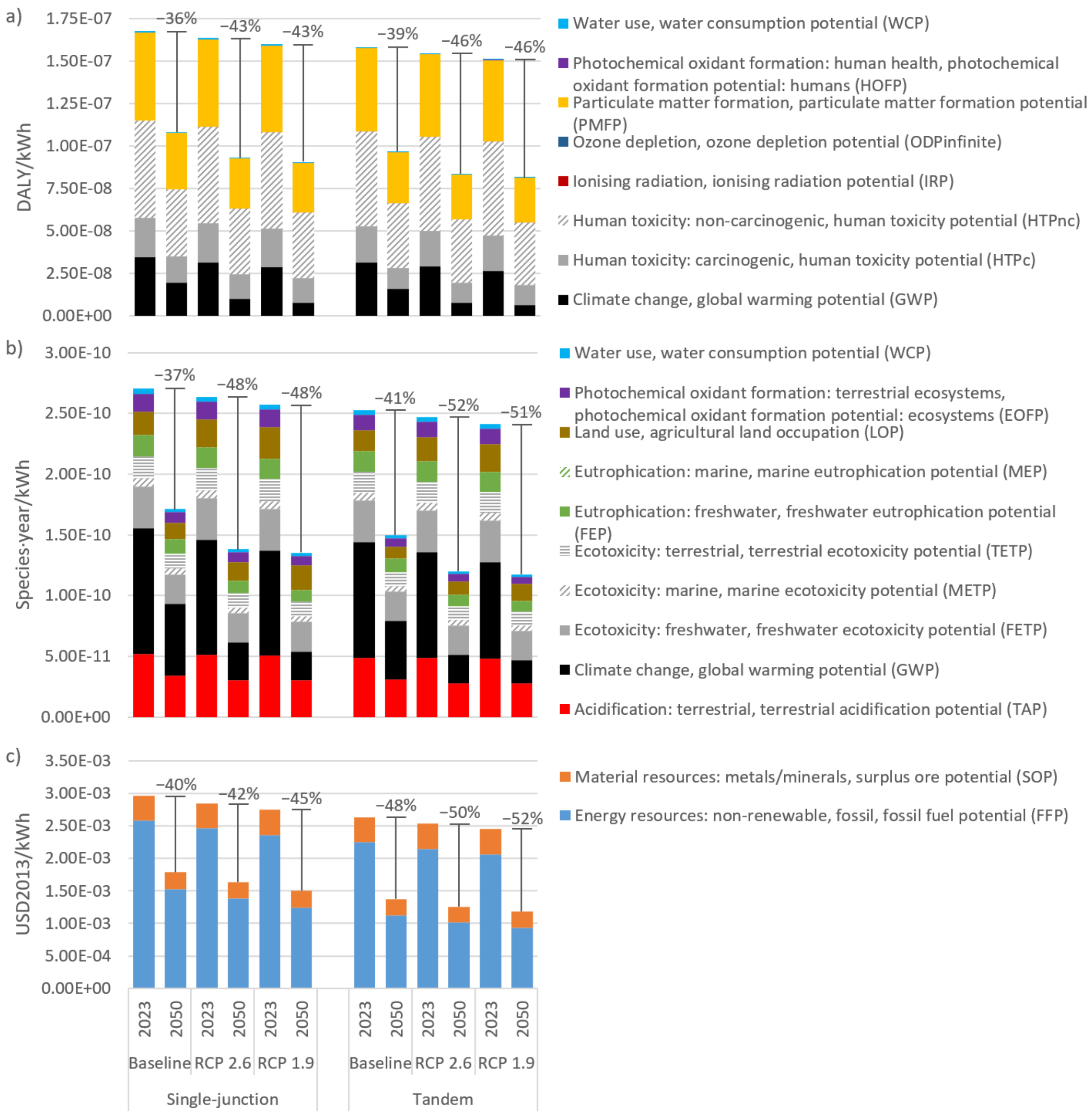

*__Figure S. 4.__ Midpoint-to-endpoint contribution analysis for the endpoints of (a) Human Health in DALY/kWh, (b) Ecosystem Quality in species*yr/kWh, and (c) Resource Scarcity in USD2013/kWh of the hierarchist (H) perspective of the ReCiPe 2016 life cycle impact assessment method. The percentages represent the decrease in impact between 2023 and 2050 for each respective background scenario. These background scenarios were based on Shared Socio-economic Pathway 2 (SSP2), i.e. the "Middle-of-the-Road" development scenario with social, economic and technological developments following historic trends. From this scenario, the baseline scenario and the Representative Concentration Pathways (RCPs) 2.6 and 1.9 were assessed, which correspond with ~3.5°C, 1.6–1.8°C and 1.2–1.4°C global mean surface temperature increase by 2100, respectively. Other assumptions: insolation: 1391 kWh/m²/yr; geographic scope: China (production) and Europe (use and end-of-life); panel design: bifacial; annual degradation rate for perovskite: 0.5% (relative). Results for the endpoints of Ecosystem Quality and Human Health are provided in the main text, __Error! Reference source not found.__.*

5.3. Midpoint-to-endpoint contribution analysis – monofacial panel – ReCiPe 2016 Individualist (I)

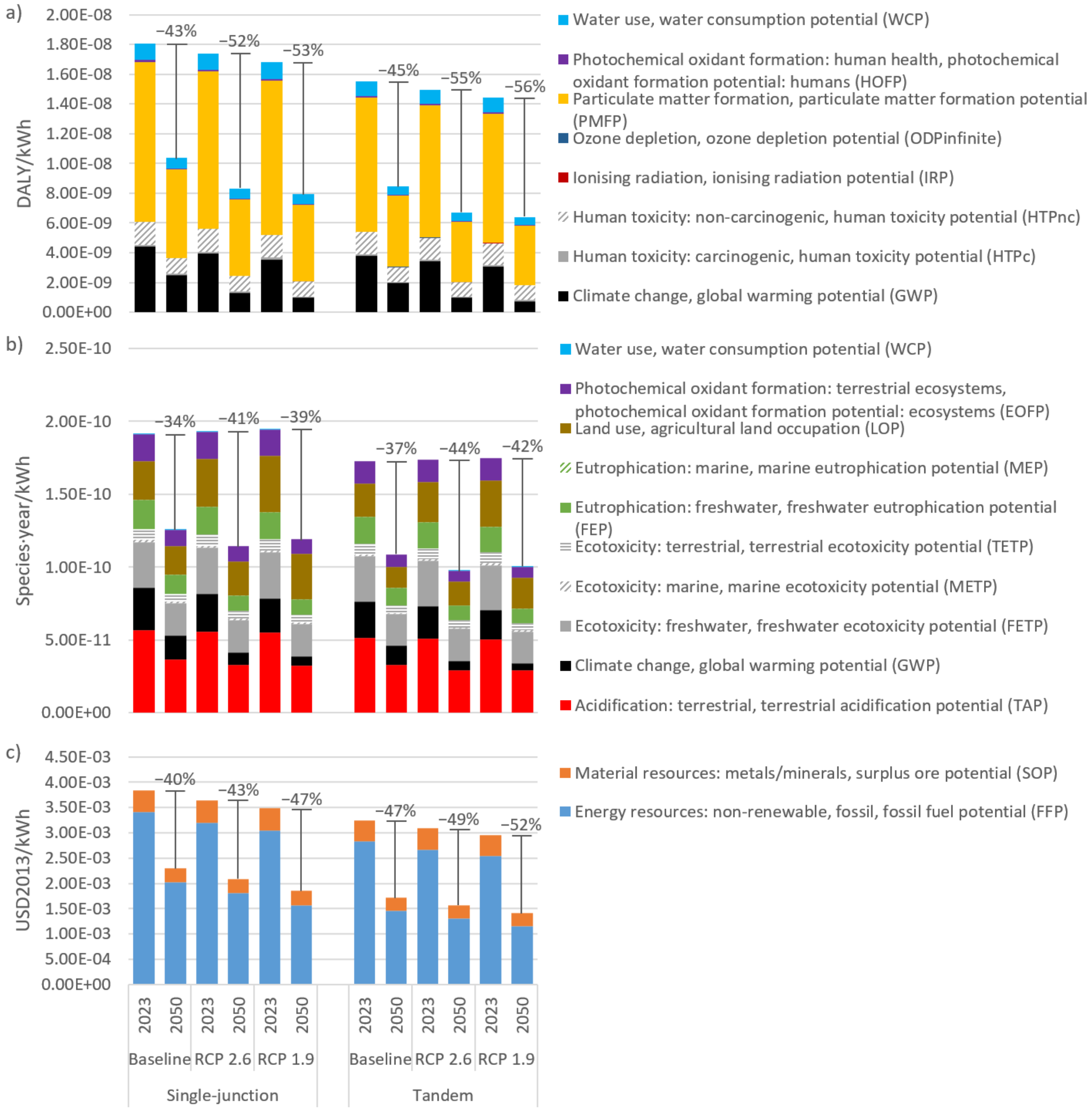

*Figure S. 5. Midpoint-to-endpoint contribution analysis for the endpoints of (a) Human Health in DALY/kWh, (b) Ecosystem Quality in species*yr/kWh, and (c) Resource Scarcity in USD2013/kWh of the individualist (I) perspective of the ReCiPe 2016 life cycle impact assessment method. The percentages represent the decrease in impact between 2023 and 2050 for each respective background scenario. These background scenarios were based on Shared Socio-economic Pathway 2 (SSP2), i.e. the "Middle-of-the-Road" development scenario with social, economic and technological developments following historic trends. From this scenario, the baseline scenario and the Representative Concentration Pathways (RCPs) 2.6 and 1.9 were assessed, which correspond with ~3.5°C, 1.6–1.8°C and 1.2–1.4°C global mean surface temperature increase by 2100, respectively. Other assumptions: insolation: 1391 kWh/m^2/yr; geographic scope: China (production) and Europe (use and end-of-life); panel design: monofacial; annual degradation rate for perovskite: 0.5% (relative).*

5.4. Midpoint-to-endpoint contribution analysis – monofacial panel – ReCiPe 2016 Egalitarian (E)

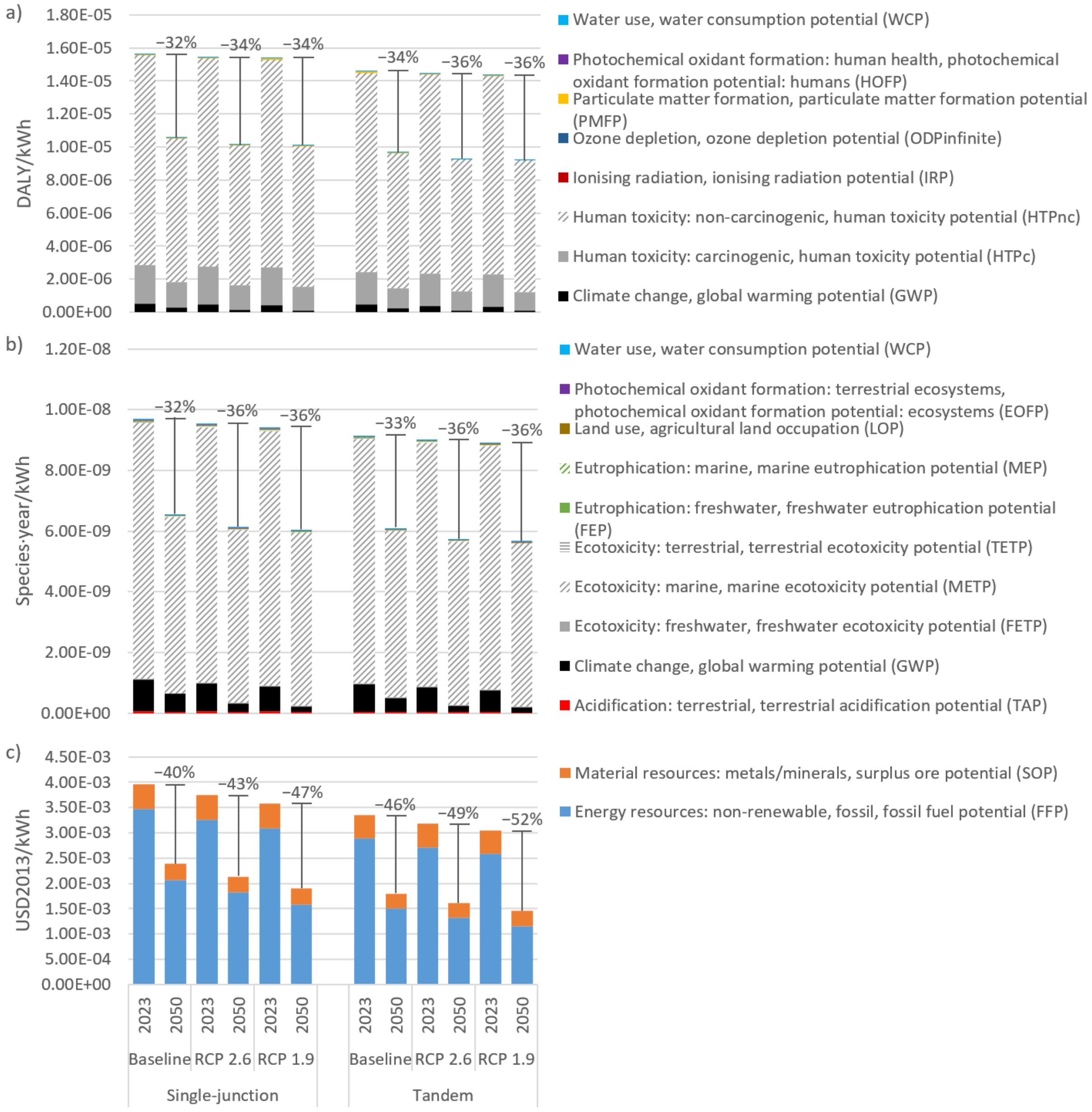

Figure S. 6. *Midpoint-to-endpoint contribution analysis for the endpoints of (a) Human Health in DALY/kWh, (b) Ecosystem Quality in species*yr/kWh, and (c) Resources Scarcity in USD2013/kWh of the individualist (I) perspective of the ReCiPe 2016 life cycle impact assessment method. The percentages represent the decrease in impact between 2023 and 2050 for each respective background scenario. These background scenarios were based on Shared Socio-economic Pathway 2 (SSP2), i.e. the "Middle-of-the-Road" development scenario with social, economic and technological developments following historic trends. From this scenario, the baseline scenario and the Representative Concentration Pathways (RCPs) 2.6 and 1.9 were assessed, which correspond with ~3.5°C, 1.6–1.8°C and 1.2–1.4°C global mean surface temperature increase by 2100, respectively. Other assumptions: insolation: 1391 kWh/m^2/yr; geographic scope: China (production) and Europe (use and end-of-life); panel design: monofacial; annual degradation rate for perovskite: 0.5% (relative).*

5.5. Midpoint-to-endpoint contribution analysis – bifacial panel – ReCiPe 2016 Individualist (I)

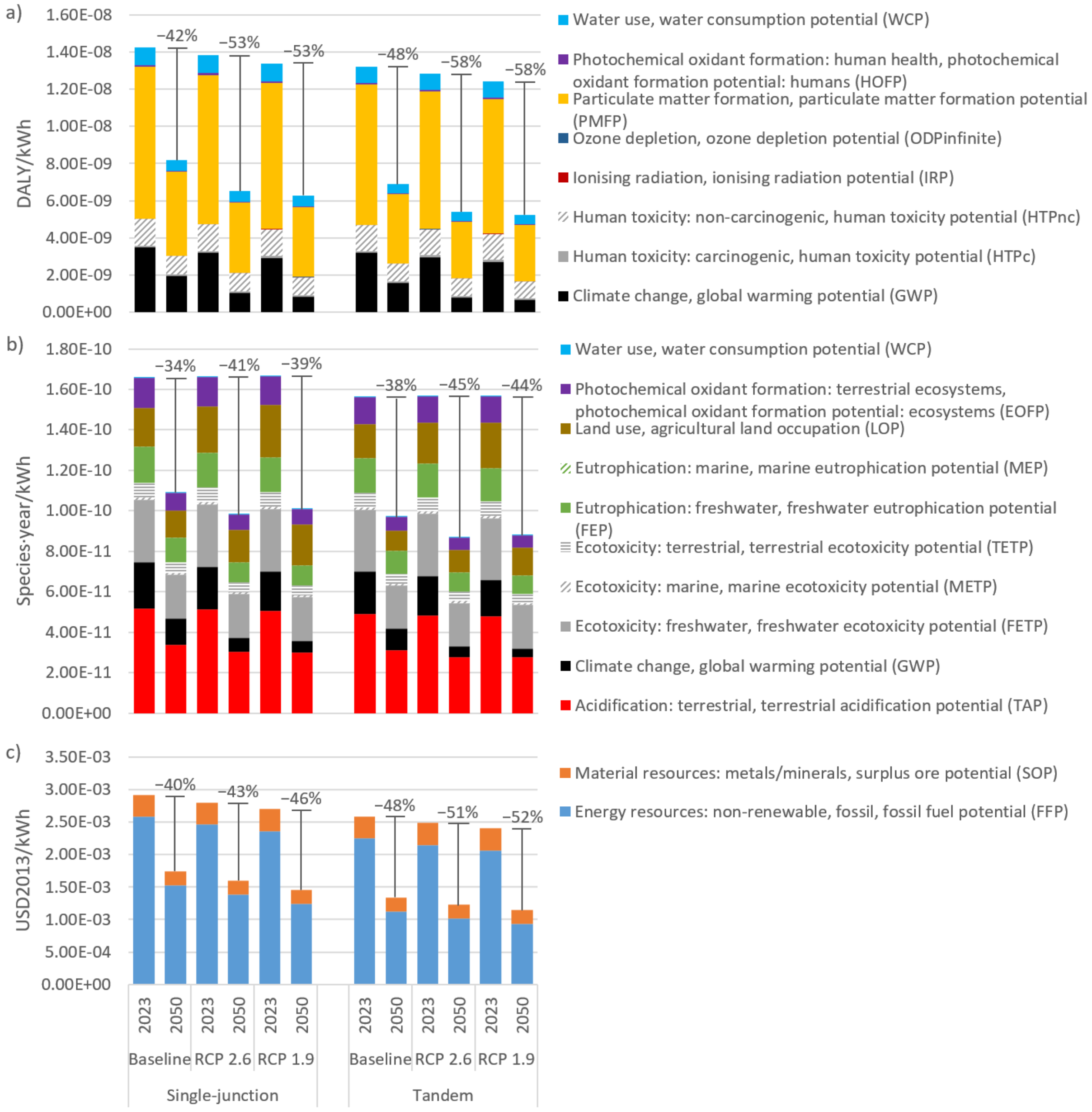

Figure S. 7. *Midpoint-to-endpoint contribution analysis for the endpoints of (a) Human Health in DALY/kWh, (b) Ecosystem Quality in species*yr/kWh, and (c) Resources Scarcity in USD2013/kWh of the individualist (I) perspective of the ReCiPe 2016 life cycle impact assessment method. The percentages represent the decrease in impact between 2023 and 2050 for each respective background scenario. These background scenarios were based on Shared Socio-economic Pathway 2 (SSP2), i.e. the "Middle-of-the-Road" development scenario with social, economic and technological developments following historic trends. From this scenario, the baseline scenario and the Representative Concentration Pathways (RCPs) 2.6 and 1.9 were assessed, which correspond with ~3.5°C, 1.6–1.8°C and 1.2–1.4°C global mean surface temperature increase by 2100, respectively. Other assumptions: insolation: 1391 kWh/m^2/yr; geographic scope: China (production) and Europe (use and end-of-life); panel design: bifacial; annual degradation rate for perovskite: 0.5% (relative).*

5.6. Midpoint-to-endpoint contribution analysis – bifacial panel – ReCiPe 2016 Egalitarian (E)

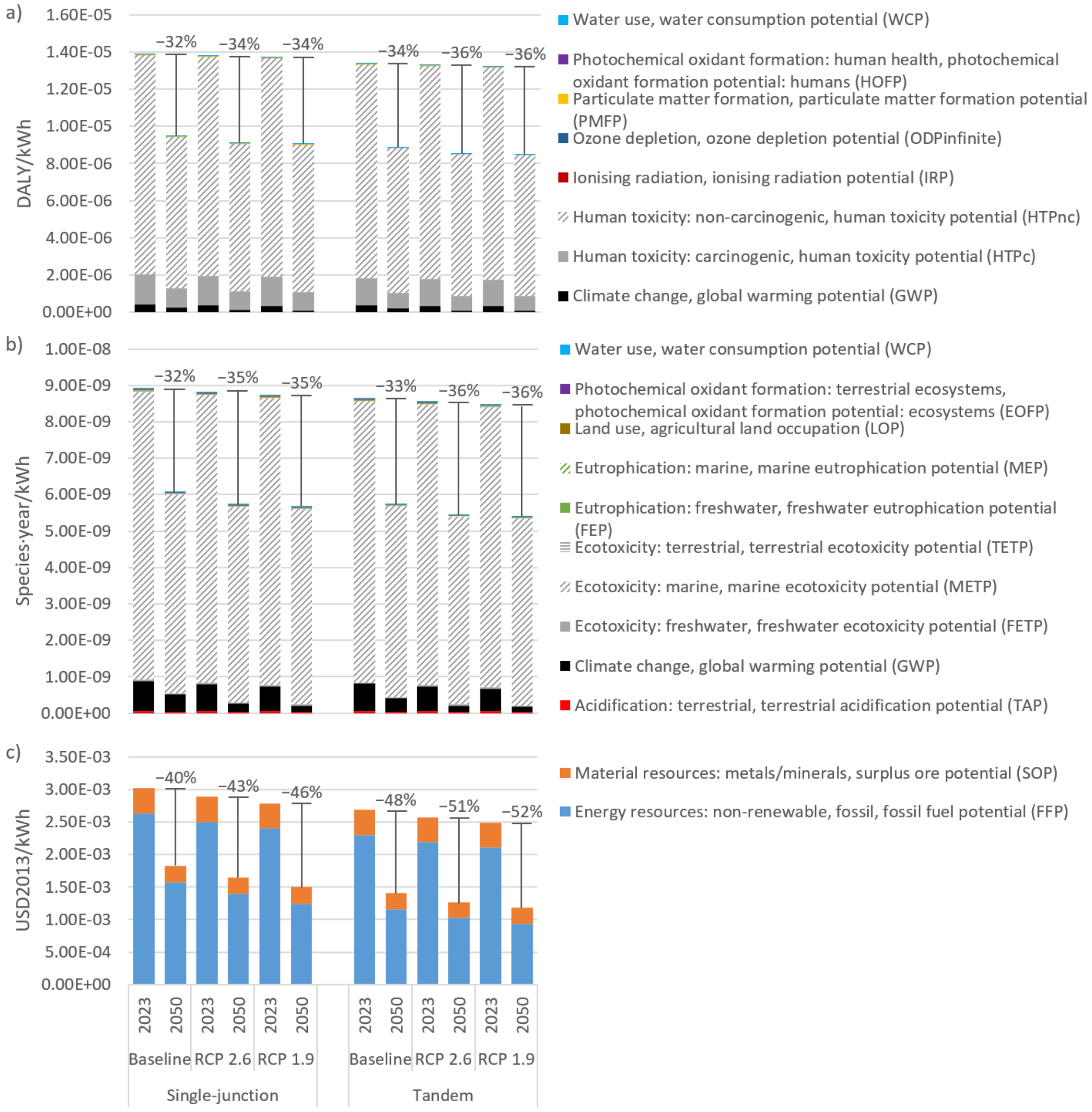

Figure S. 8. *Midpoint-to-endpoint contribution analysis for the endpoints of (a) Human Health in DALY/kWh, (b) Ecosystem Quality in species*yr/kWh, and (c) Resources Scarcity in USD2013/kWh of the egalitarian (E) perspective of the ReCiPe 2016 life cycle impact assessment method. The percentages represent the decrease in impact between 2023 and 2050 for each respective background scenario. These background scenarios were based on Shared Socio-economic Pathway 2 (SSP2), i.e. the "Middle-of-the-Road" development scenario with social, economic and technological developments following historic trends. From this scenario, the baseline scenario and the Representative Concentration Pathways (RCPs) 2.6 and 1.9 were assessed, which correspond with ~3.5°C, 1.6–1.8°C and 1.2–1.4°C global mean surface temperature increase by 2100, respectively. Other assumptions: insolation: 1391 kWh/m^2/yr; geographic scope: China (production) and Europe (use and end-of-life); panel design: bifacial; annual degradation rate for perovskite: 0.5% (relative).*

5.7. Climate Change – SSP2-Base – EF v3.1

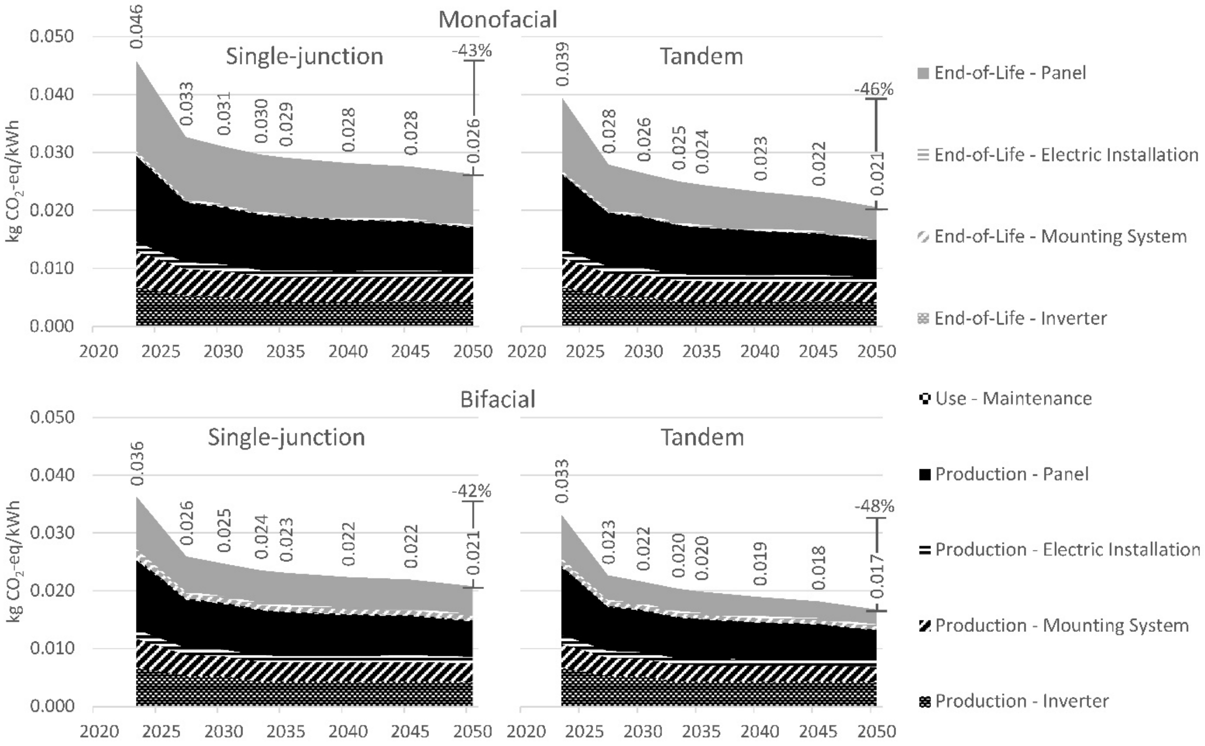

Figure S. 9. *Process contribution analysis for the impact category of Climate Change in kg CO_2-eq./kWh using the EF v3.1 method. The x-axis represent the year of production for the panel. Impacts from end-of-life were modelled to occur after the economic lifespan of the panel. For example, end-of-life for the panel produced in 2023 was modelled to occur in 2053. Assumptions: panel design: monofacial; insolation: 1391 kWh/m²/yr; geographic scope: China (production) and Europe (use and end-of-life); annual degradation rate for perovskite: 0.5% (relative); background scenario: Shared Socio-economic Pathway 2 - baseline (corresponding with 3–4°C global mean surface temperature increase by 2100).*

5.8. Climate Change – SSP2-RCP1.9 – EF v3.1

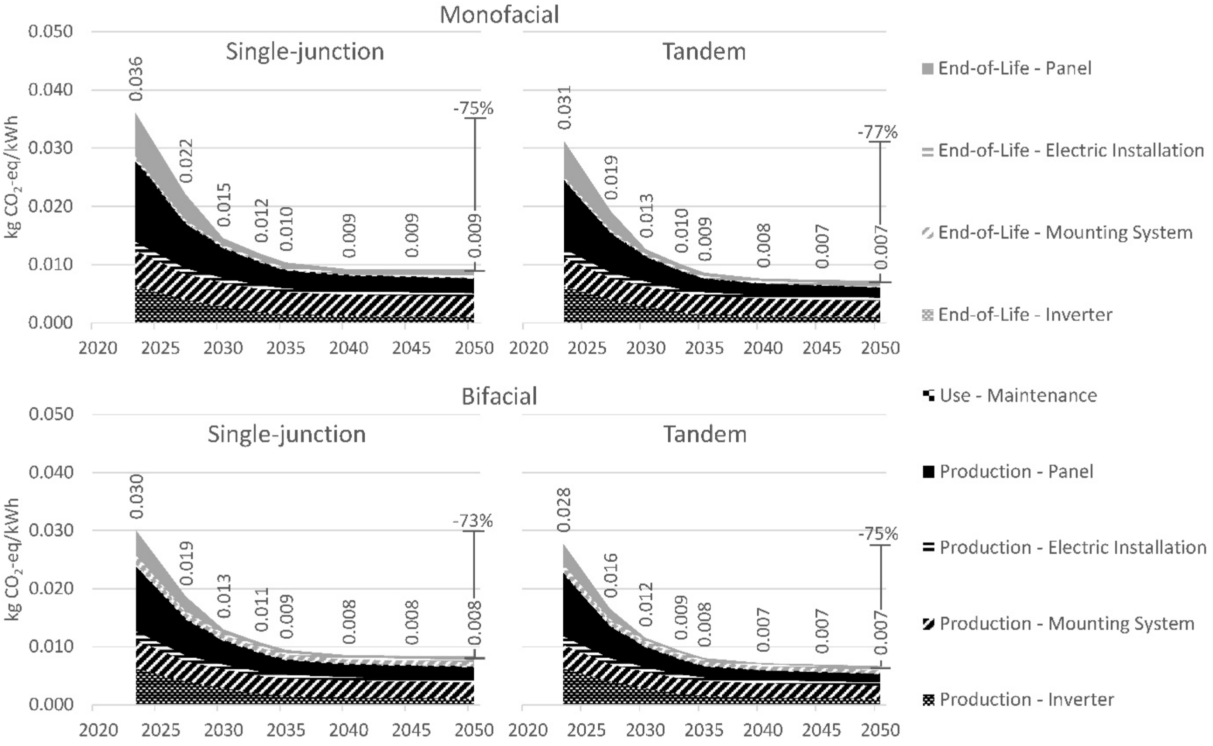

Figure S. 10. *Process contribution analysis for the impact category of Climate Change in kg CO_2-eq./kWh using the EF v3.1 method. The x-axis represent the year of production for the panel. Impacts from end-of-life were modelled to occur after the economic lifespan of the panel. For example, end-of-life for the panel produced in 2023 was modelled to occur in 2053. Assumptions: panel design: monofacial; insolation: 1391 kWh/m^2/yr; geographic scope: China (production) and Europe (use and end-of-life); annual degradation rate for perovskite: 0.5% (relative); background scenario: Shared Socio-economic Pathway 2 - Representative Concentration Pathway 1.9 (corresponding with 1.2–1.4°C global mean surface temperature increase by 2100).*

5.9. Climate Change – SSP2-base – ReCiPe 2016 Hierarchist (H)

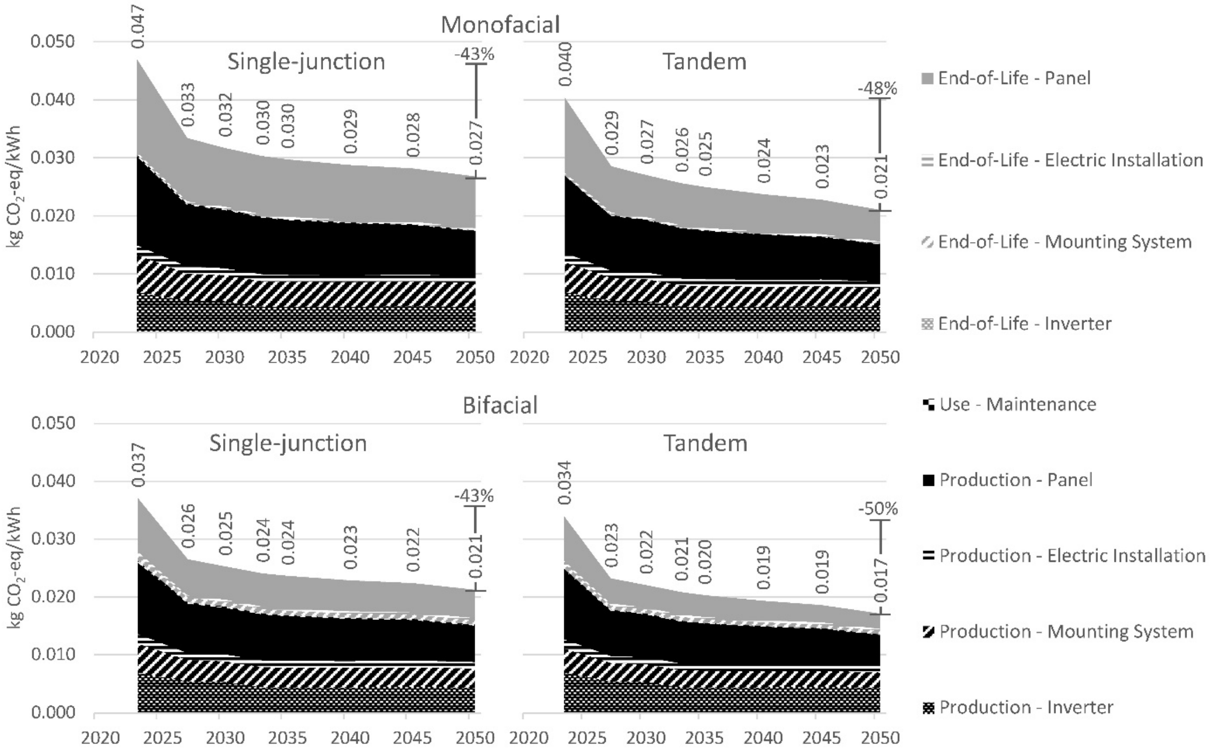

Figure S. 11. *Process contribution analysis for the impact category of Climate Change in kg CO_2-eq./kWh using the hierarchist (H) perspective of the ReCiPe 2016 LCIA method. The x-axis represent the year of production for the panel. Impacts from end-of-life were modelled to occur after the economic lifespan of the panel. For example, end-of-life for the panel produced in 2023 was modelled to occur in 2053. Assumptions: panel design: monofacial; insolation: 1391 kWh/m^2/yr; geographic scope: China (production) and Europe (use and end-of-life); annual degradation rate for perovskite: 0.5% (relative); background scenario: Shared Socio-economic Pathway 2 - baseline (corresponding with 3–4°C global mean surface temperature increase by 2100).*

5.10. Climate Change – SSP2-RCP2.6– ReCiPe 2016 Hierarchist (H)

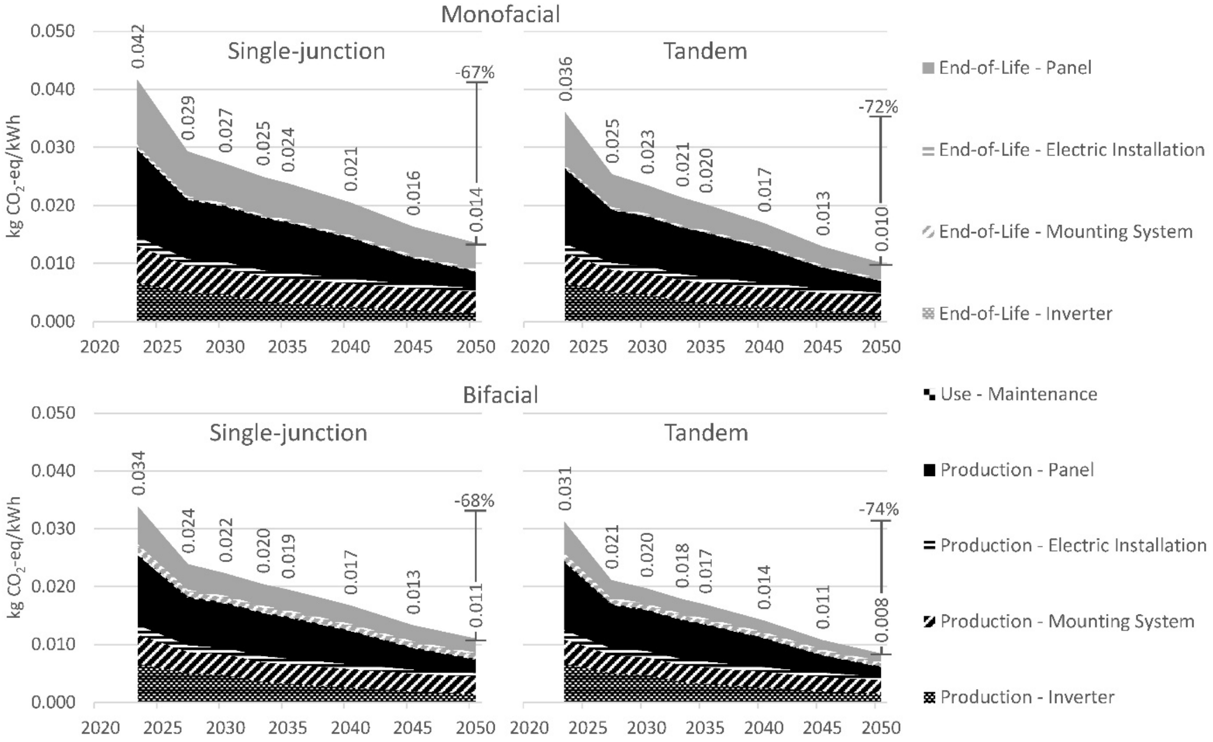

Figure S. 12. *Process contribution analysis for the impact category of Climate Change in kg CO_2-eq./kWh using the hierarchist (H) perspective of the ReCiPe 2016 LCIA method. The x-axis represent the year of production for the panel. Impacts from end-of-life were modelled to occur after the economic lifespan of the panel. For example, end-of-life for the panel produced in 2023 was modelled to occur in 2053. Assumptions: panel design: monofacial; insolation: 1391 kWh/m^2/yr; geographic scope: China (production) and Europe (use and end-of-life); annual degradation rate for perovskite: 0.5% (relative); background scenario: Shared Socio-economic Pathway 2 - Representative Concentration Pathway 2.6 (corresponding with 1.6–1.8°C global mean surface temperature increase by 2100).*

5.11. Climate Change – SSP2-RCP1.9 – ReCiPe 2016 Hierarchist (H)

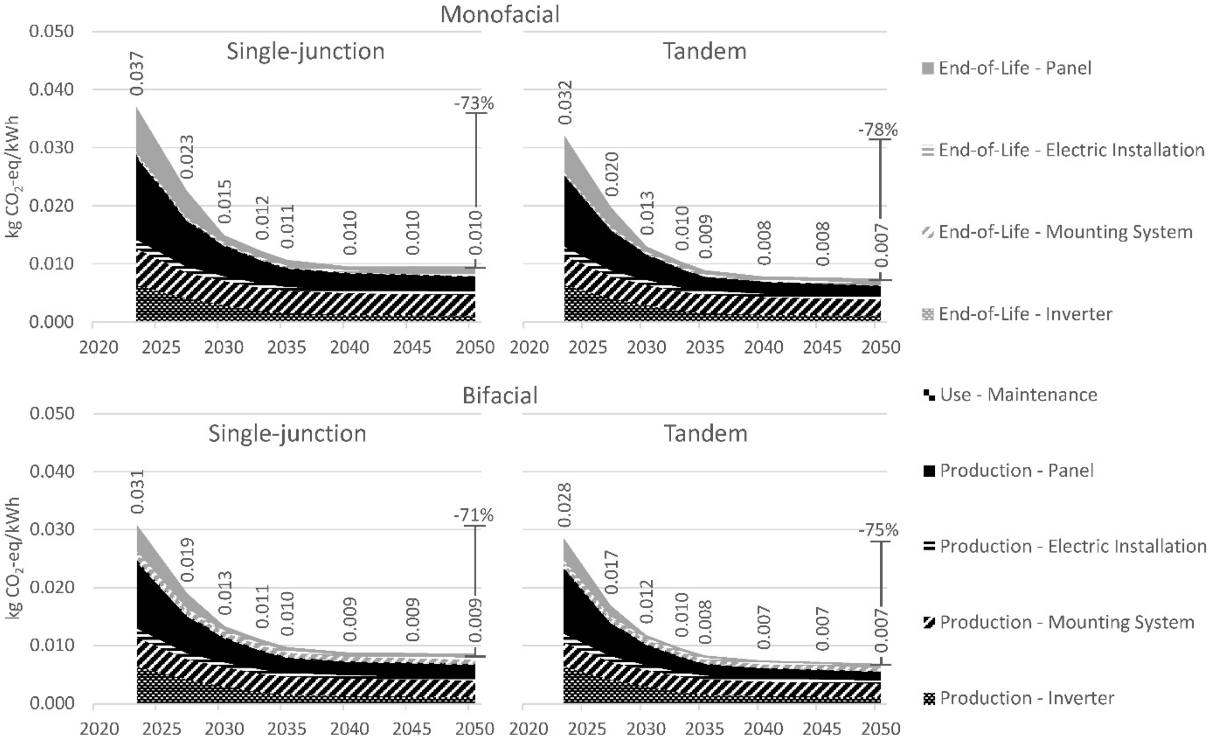

Figure S. 13. *Process contribution analysis for the impact category of Climate Change in kg CO_2-eq./kWh using the hierarchist (H) perspective of the ReCiPe 2016 LCIA method. The x-axis represent the year of production for the panel. Impacts from end-of-life were modelled to occur after the economic lifespan of the panel. For example, end-of-life for the panel produced in 2023 was modelled to occur in 2053. Assumptions: panel design: monofacial; insolation: 1391 kWh/m^2/yr; geographic scope: China (production) and Europe (use and end-of-life); annual degradation rate for perovskite: 0.5% (relative); background scenario: Shared Socio-economic Pathway 2 - Representative Concentration Pathway 1.9 (corresponding with 1.2–1.4°C global mean surface temperature increase by 2100).*

5.12. Climate Change – SSP2-base – ReCiPe 2016 Individualist (I)

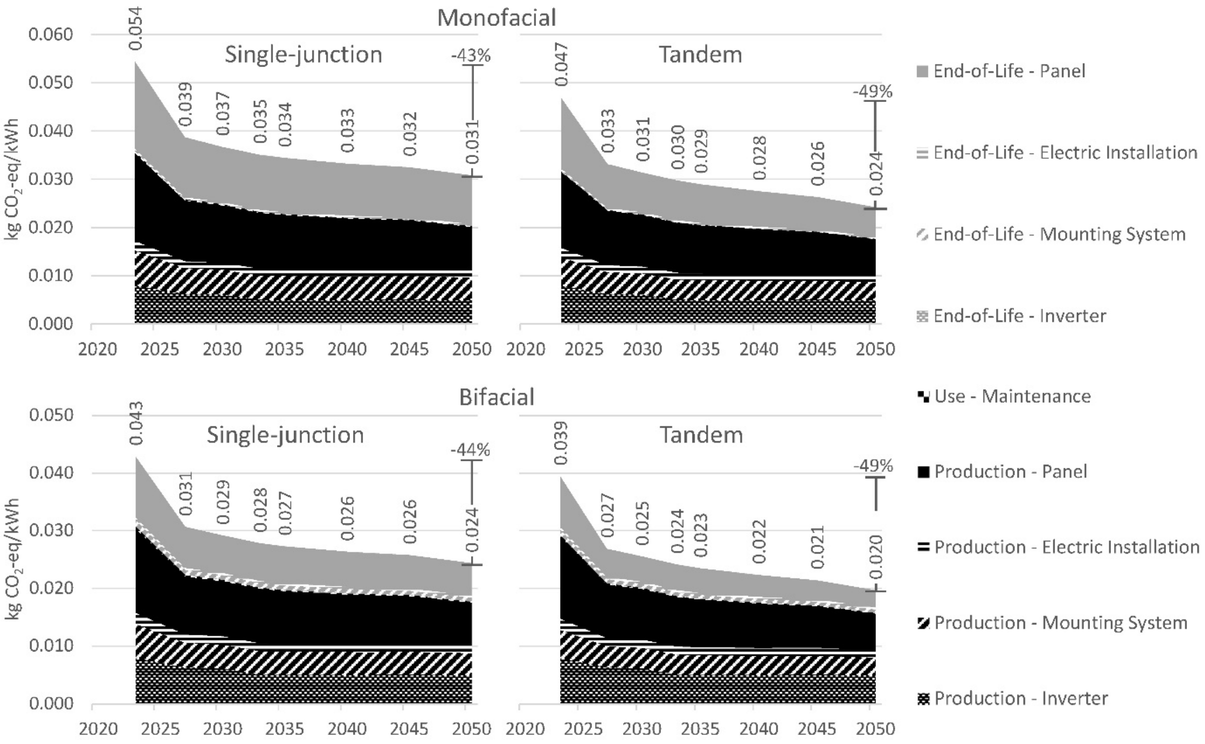

Figure S. 14. *Process contribution analysis for the impact category of Climate Change in kg CO_2-eq./kWh using the individualist (I) perspective of the ReCiPe 2016 LCIA method. Note that the y-axis for the mono-facial and bifacial panels have different scales. The x-axis represent the year of production for the panel. Impacts from end-of-life were modelled to occur after the economic lifespan of the panel. For example, end-of-life for the panel produced in 2023 was modelled to occur in 2053. Assumptions: panel design: monofacial; insolation: 1391 kWh/m^2/yr; geographic scope: China (production) and Europe (use and end-of-life); annual degradation rate for perovskite: 0.5% (relative); background scenario: Shared Socio-economic Pathway 2 - baseline (corresponding with 3–4°C global mean surface temperature increase by 2100).*

5.13. Climate Change – SSP2-RCP2.6 – ReCiPe 2016 Individualist (I)

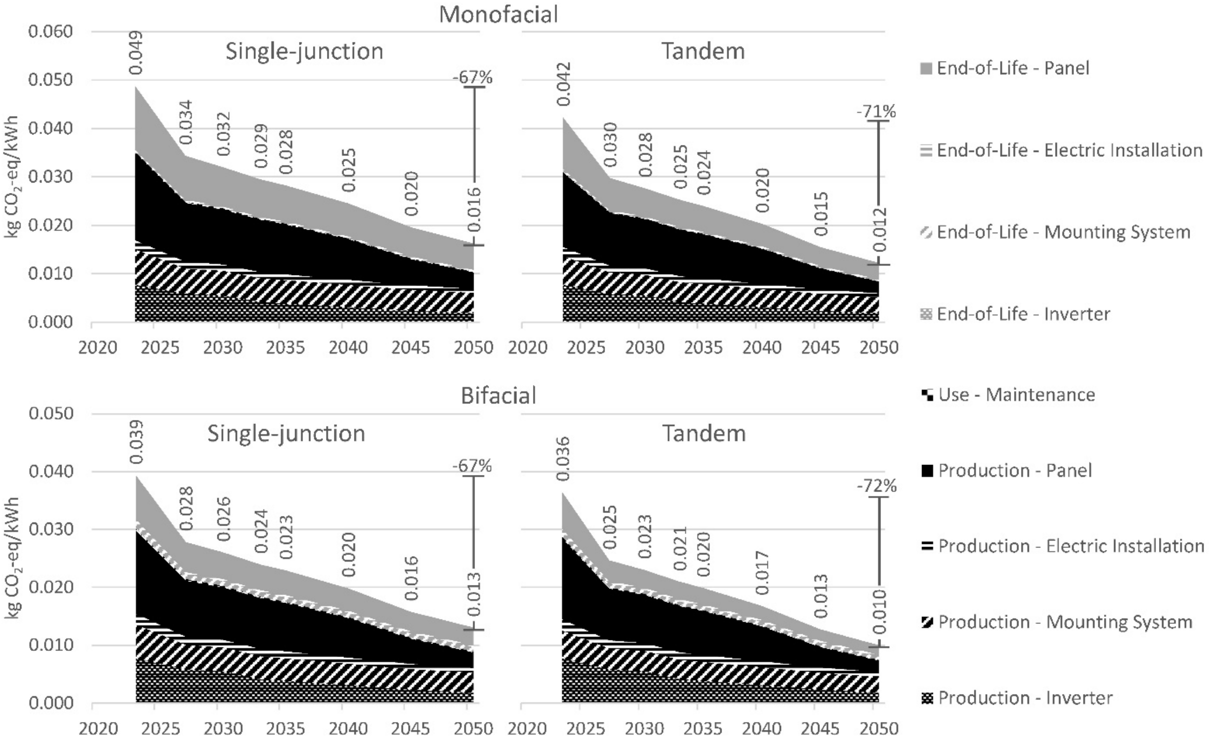

Figure S. 15. *Process contribution analysis for the impact category of Climate Change in kg CO_2-eq./kWh using the individualist (I) perspective of the ReCiPe 2016 LCIA method. Note that the y-axis for the mono-facial and bifacial panels have different scales. The x-axis represent the year of production for the panel. Impacts from end-of-life were modelled to occur after the economic lifespan of the panel. For example, end-of-life for the panel produced in 2023 was modelled to occur in 2053. Assumptions: panel design: monofacial; insolation: 1391 kWh/m^2/yr; geographic scope: China (production) and Europe (use and end-of-life); annual degradation rate for perovskite: 0.5% (relative); background scenario: Shared Socio-economic Pathway 2 - Representative Concentration Pathway 2.6 (corresponding with 1.6–1.8°C global mean surface temperature increase by 2100).*

5.14. Climate Change – SSP2-RCP1.9 – ReCiPe 2016 Individualist (I)

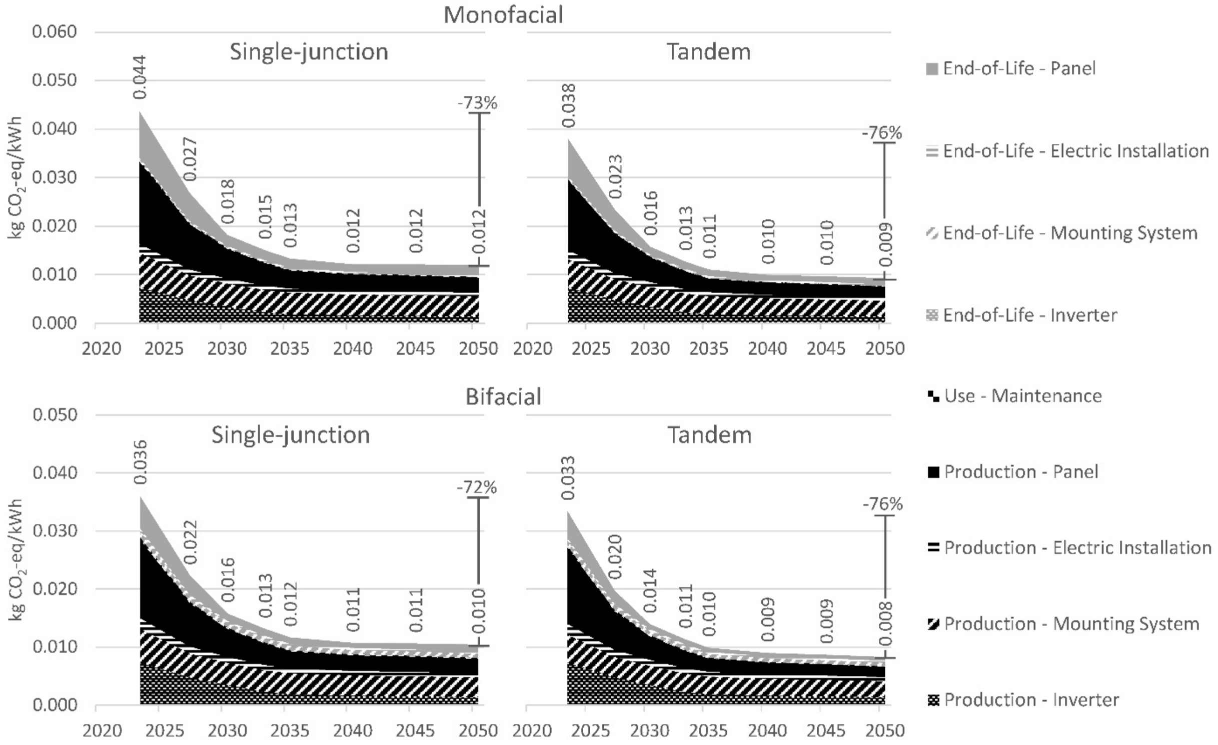

Figure S. 16. *Process contribution analysis for the impact category of Climate Change in kg CO_2-eq./kWh using the individualist (I) perspective of the ReCiPe 2016 LCIA method. The x-axis represent the year of production for the panel. Impacts from end-of-life were modelled to occur after the economic lifespan of the panel. For example, end-of-life for the panel produced in 2023 was modelled to occur in 2053. Assumptions: panel design: monofacial; insolation: 1391 kWh/m^2/yr; geographic scope: China (production) and Europe (use and end-of-life); annual degradation rate for perovskite: 0.5% (relative); background scenario: Shared Socio-economic Pathway 2 - Representative Concentration Pathway 1.9 (corresponding with 1.2–1.4°C global mean surface temperature increase by 2100).*

5.15. Climate Change – SSP2-base – ReCiPe 2016 Egalitarian (E)

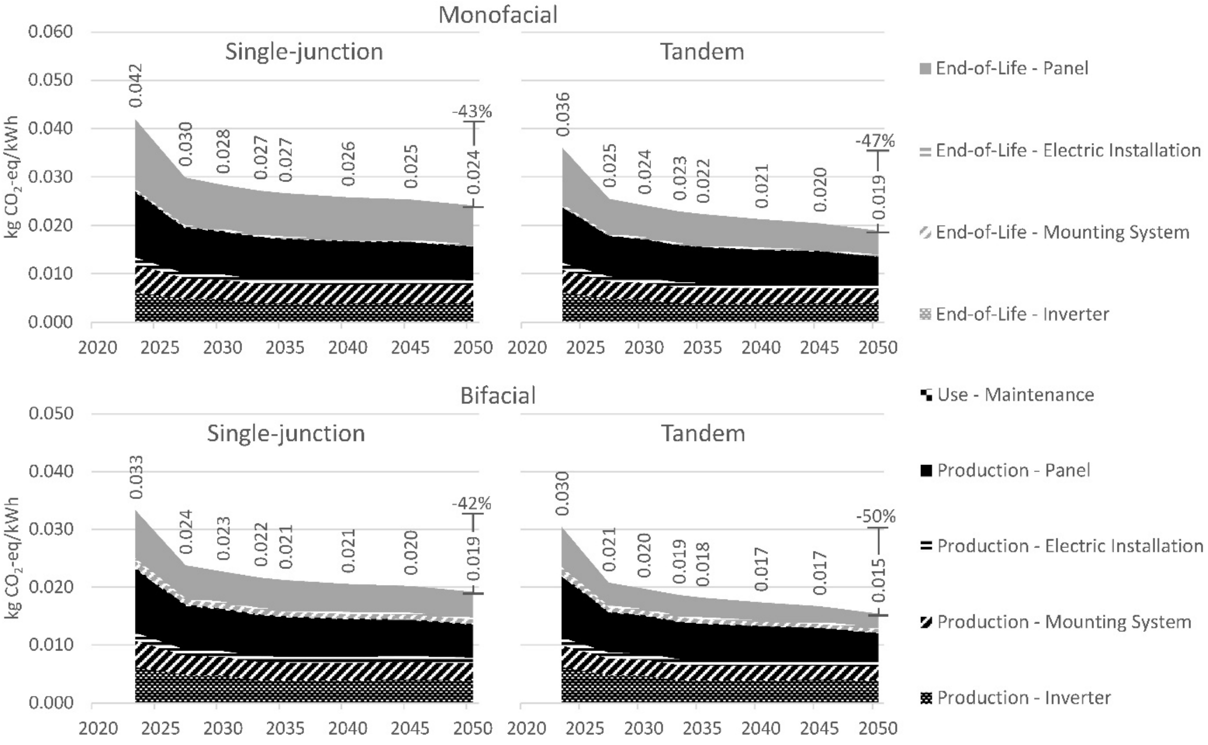

__Figure S. 17.__ Process contribution analysis for the impact category of Climate Change in kg CO_2-eq./kWh using the egalitarian (E) perspective of the ReCiPe 2016 LCIA method. The x-axis represent the year of production for the panel. Impacts from end-of-life were modelled to occur after the economic lifespan of the panel. For example, end-of-life for the panel produced in 2023 was modelled to occur in 2053. Assumptions: panel design: monofacial; insolation: 1391 kWh/m^2/yr; geographic scope: China (production) and Europe (use and end-of-life); annual degradation rate for perovskite: 0.5% (relative); background scenario: Shared Socio-economic Pathway 2 - baseline (corresponding with 3–4°C global mean surface temperature increase by 2100).

5.16. Climate Change – SSP2-RCP2.6 – ReCiPe 2016 Egalitarian (E)

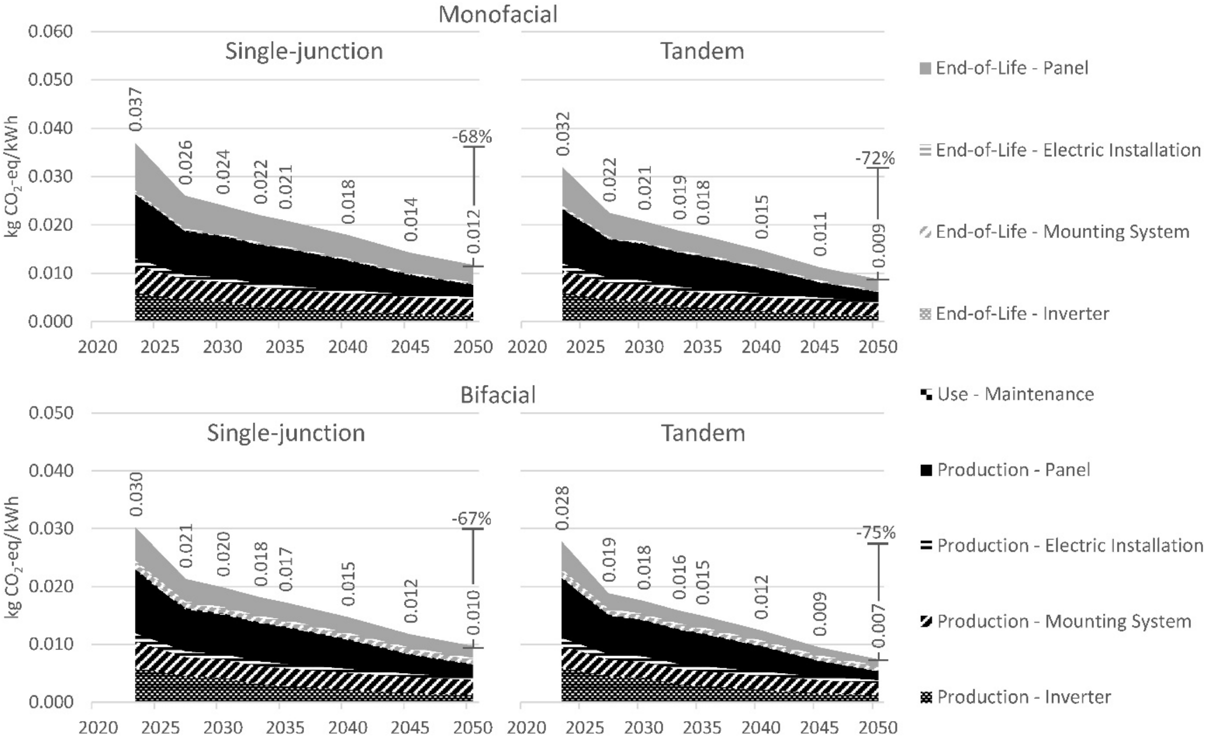

Figure S. 18. *Process contribution analysis for the impact category of Climate Change in kg CO_2-eq./kWh using the egalitarian (E) perspective of the ReCiPe 2016 LCIA methodThe x-axis represent the year of production for the panel. Impacts from end-of-life were modelled to occur after the economic lifespan of the panel. For example, end-of-life for the panel produced in 2023 was modelled to occur in 2053. Assumptions: panel design: monofacial; insolation: 1391 kWh/m^2/yr; geographic scope: China (production) and Europe (use and end-of-life); annual degradation rate for perovskite: 0.5% (relative); background scenario: Shared Socio-economic Pathway 2 - Representative Concentration Pathway 2.6 (corresponding with 1.6–1.8°C global mean surface temperature increase by 2100).*

5.17. Climate Change – SSP2-RCP1.9 – ReCiPe 2016 Egalitarian (E)

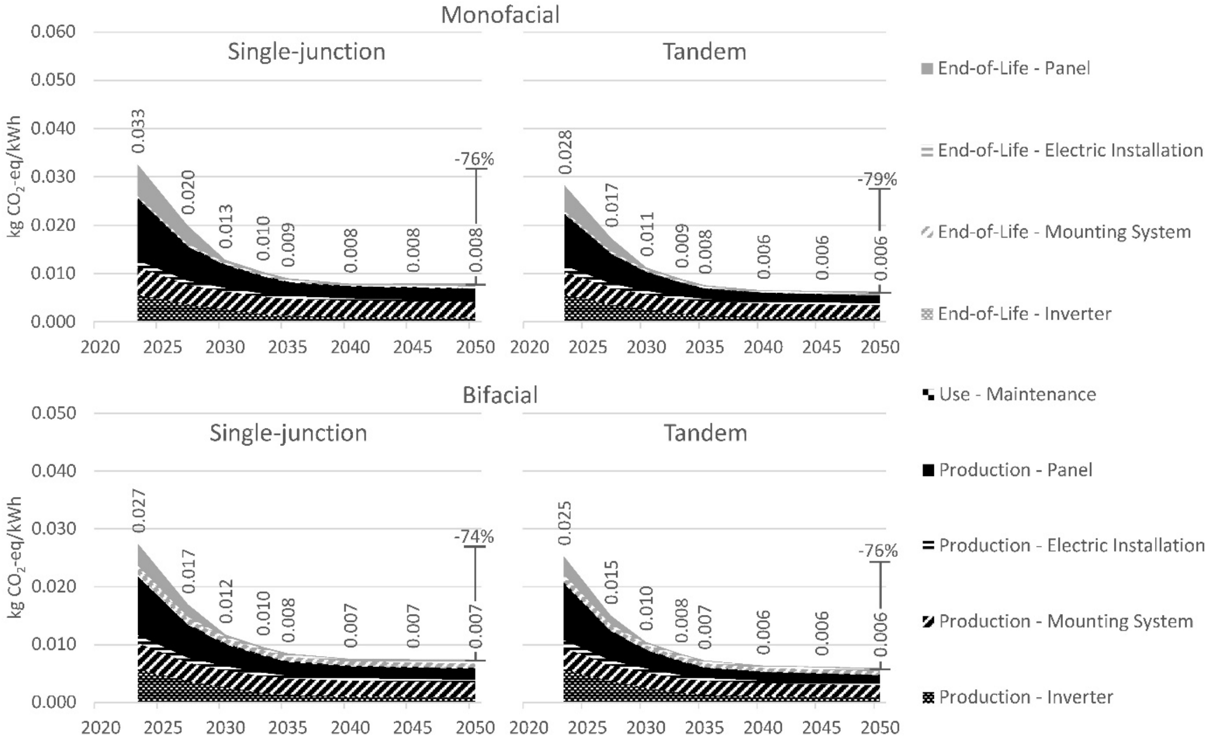

Figure S. 19. *Process contribution analysis for the impact category of Climate Change in kg CO_2-eq./kWh using the egalitarian (E) perspective of the ReCiPe 2016 LCIA method. The x-axis represent the year of production for the panel. Impacts from end-of-life were modelled to occur after the economic lifespan of the panel. For example, end-of-life for the panel produced in 2023 was modelled to occur in 2053. Assumptions: panel design: monofacial; insolation: 1391 kWh/m^2/yr; geographic scope: China (production) and Europe (use and end-of-life); annual degradation rate for perovskite: 0.5% (relative); background scenario: Shared Socio-economic Pathway 2 - Representative Concentration Pathway 1.9 (corresponding with 1.2–1.4°C global mean surface temperature increase by 2100).*

5.18. Climate Change – SSP2-RCP2.6 – EF v3.1 – No time lag

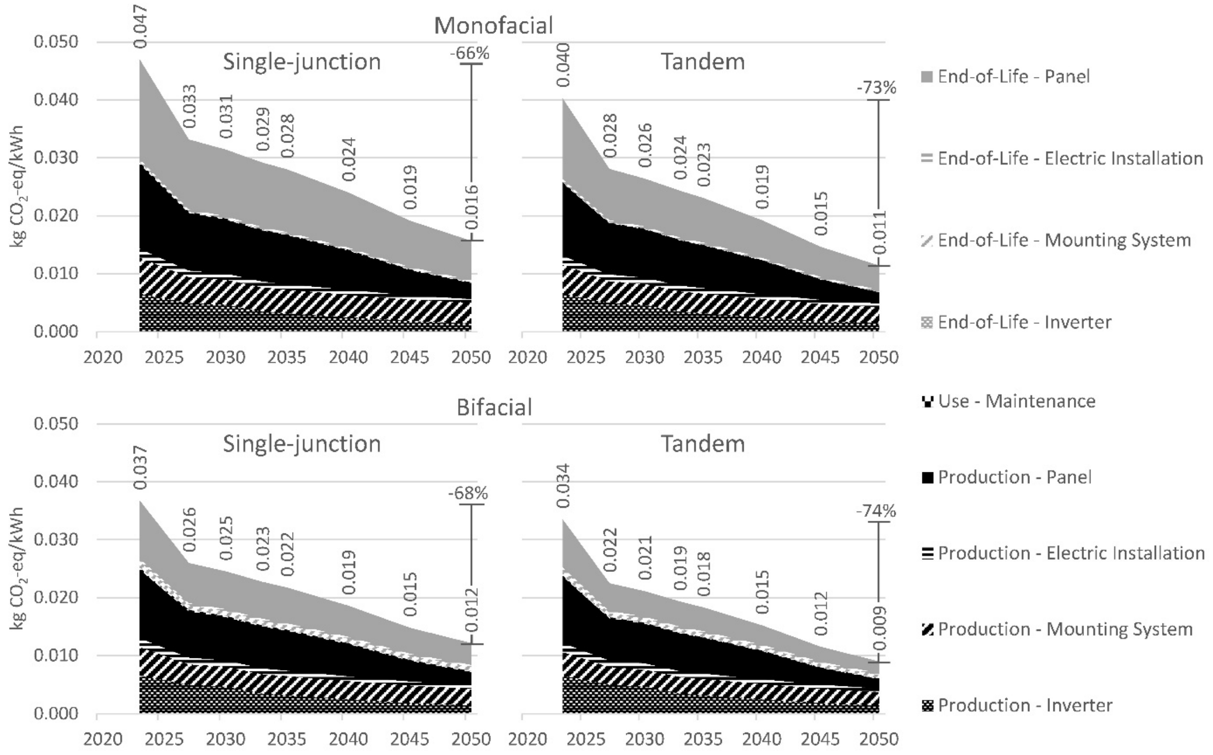

Figure S. 20. *Process contribution analysis for the impact category of Climate Change in kg CO_2-eq./kWh. For end-of-life, impacts were modelled to occur at the same time as panel production. For example, end-of-life for the panel produced in 2023 was modelled to also occur in 2023. Assumptions: insolation: 1391 kWh/m²/yr; geographic scope: China (production) and Europe (use and end-of-life); annual degradation rate for perovskite: 0.5% (relative); background scenario: IMAGE SSP2 RCP 2.6 (Shared Socio-economic Pathway 2 - Representative Concentration Pathway 2.6, corresponding with 1.6–1.8°C global mean surface temperature increase by 2100).*

5.19. Climate Change – SSP2-RCP2.6 – EF v3.1 – Increased annual degradation rates

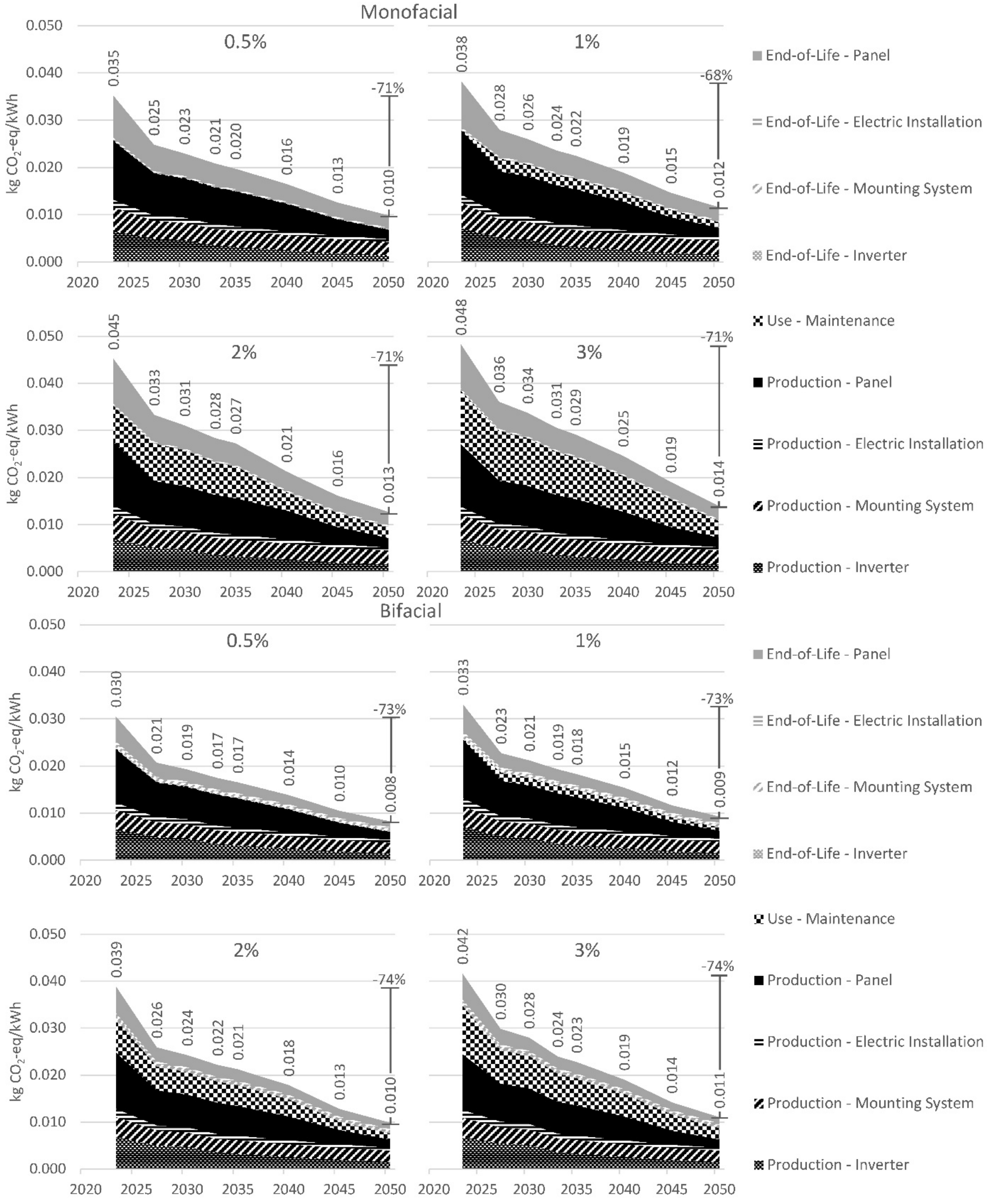

Figure S. 21. *Process contribution analysis for the impact category of Climate Change in kg CO_2-eq./kWh using the EF v3.1 method for annual degradation rates varying from 0.5% to 3% (relative). The x-axis represent the year of production for the panel. Impacts from end-of-life were modelled to occur after the economic lifespan of the panel. For example, end-of-life for the panel produced in 2023 was modelled to occur in 2053. Assumptions: panel type: silicon/perovskite tandem; insolation: 1391 kWh/m^2/yr; geographic scope: China (production) and Europe (use and end-of-life); background scenario: Shared Socio-economic Pathway 2 - Representative Concentration Pathway 2.6 (corresponding with 1.6–1.8°C global mean surface temperature increase by 2100).*

5.20. Scenario analyses – Climate Change – EF v3.1

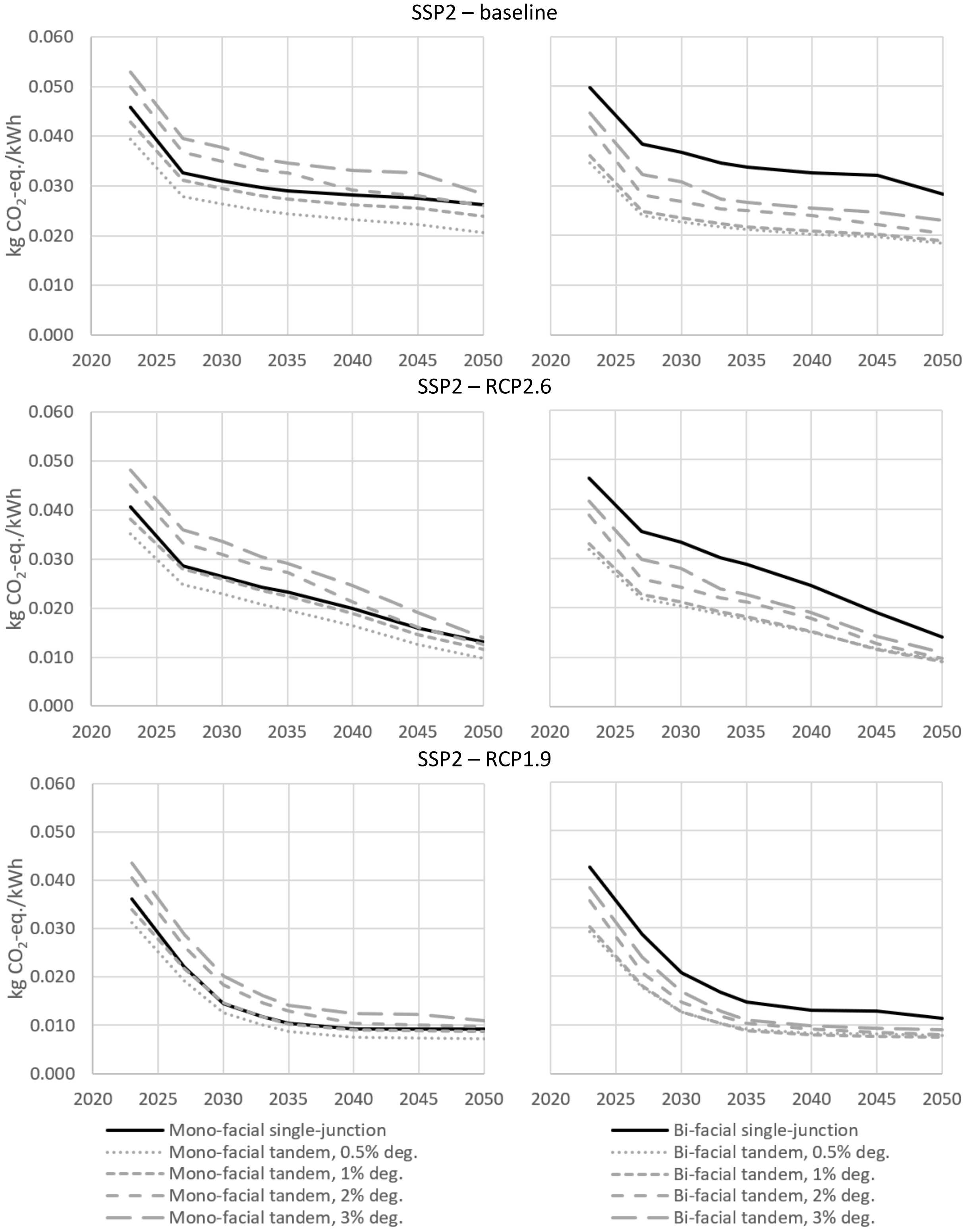

Figure S. 22. *Scenario analysis for the influence of the degradation rate of the tandem panel on the impact category of Climate Change in kg CO_2-eq./kWh using the EF v3.1 method. Background scenarios were based on Shared Socio-economic Pathway 2 (SSP2), i.e. the "Middle-of-the-Road" development scenario with social, economic and technological developments following historic trends. From this scenario, the baseline scenario and the Representative Concentration Pathways (RCPs) 2.6 and 1.9 were assessed, which correspond with ~3.5°C, 1.6–1.8°C and 1.2–1.4°C global mean surface temperature increase by 2100, respectively. Other assumptions: insolation: 1391 kWh/m^2/yr; geographic scope: China (production) and Europe (use and end-of-life).*

5.21. Variance contribution analysis – IEA PVPS

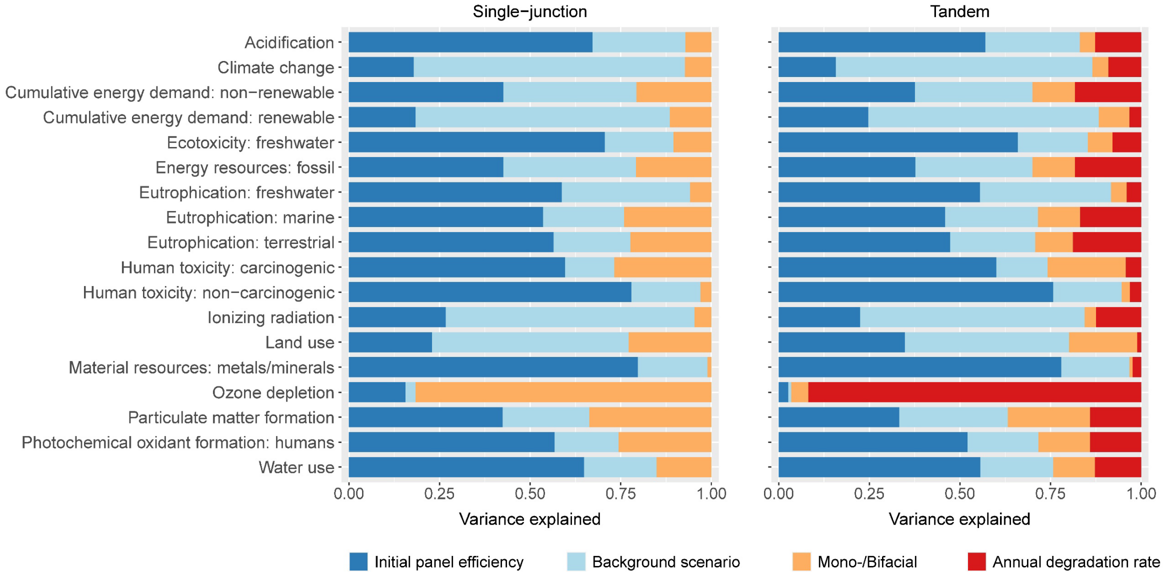

Figure S. 23. Variance decomposition analysis for the results of all midpoint impact categories for the single-junction (left) or tandem panel (right) using the set of LCIA methods recommended by the IEA PVPS.

6. REFERENCES

(1) Müller, A.; Friedrich, L.; Reichel, C.; Herceg, S.; Mittag, M.; Neuhaus, D. H. A comparative life cycle assessment of silicon PV modules: Impact of module design, manufacturing location and inventory. *Sol. Energy Mater. Sol. Cells* **2021**, *230*, 111277. DOI: 10.1016/j.solmat.2021.111277.
(2) Fraunhofer ISE. *Photovoltaics Report - 21 February 2023*; 2023. https://www.ise.fraunhofer.de/content/dam/ise/de/documents/publications/studies/Photovoltaics-Report.pdf (accessed 2023-06-09).
(3) polymerdatabase.com. *Poly(isobutene)*; polymerdatabase.com, 2022. https://polymerdatabase.com/polymers/polyisobutene.html (accessed 2022-08-16).
(4) Tsang, M. P.; Sonnemann, G. W.; Bassani, D. M. A comparative human health, ecotoxicity, and product environmental assessment on the production of organic and silicon solar cells. *Prog. Photovolt.: Res. Appl.* **2016**, *24* (5), 645–655. DOI: 10.1002/pip.2704.
(5) Alberola-Borràs, J.-A.; Vidal, R.; Mora-Seró, I. Evaluation of multiple cation/anion perovskite solar cells through life cycle assessment. *Sustain. Energy Fuels* **2018**, *2* (7), 1600–1609. DOI: 10.1039/C8SE00053K.
(6) Khalifa, S. A.; Spatari, S.; Fafarman, A. T.; Baxter, J. B. Environmental Sustainability of Mixed Cation Perovskite Materials in Photovoltaics Manufacturing. *ACS Sustain. Chem. Eng.* **2020**, *8* (44), 16537–16548. DOI: 10.1021/acssuschemeng.0c05619.
(7) Crenna, E. *Dataset Information (UPR) spodumene production, AU, (Author: Eleonora Crenna inactive)*; ecoinvent, 2021. https://v38.ecoquery.ecoinvent.org (accessed 2022-08-05).
(8) Hischier, R. *Packaging glass. Ecoinvent report No. 11-IV*; Swiss Centre for Life Cycle Inventories, Dübendorf, Switzerland, 2007. https://db.ecoinvent.org/reports/11_IV_PackagingGlass.pdf (accessed 2019-10-21).
(9) Hischier, R. *spodumene production, AU, Allocation, cut-off by classification, ecoinvent database version 3.8*; ecoinvent, 2007. https://v38.ecoquery.ecoinvent.org (accessed 2022-08-05).
(10) Tuck, C. A. *Mineral Commodity Summaries, January 2020 - Cesium*; U.S. Geological Survey, 2020. https://pubs.usgs.gov/periodicals/mcs2020/mcs2020-cesium.pdf (accessed 2022-08-05).
(11) Tuck, C. A. *Mineral Commodity Summaries, February 2019 - Cesium*; U.S. Geological Survey, 2019. https://d9-wret.s3.us-west-2.amazonaws.com/assets/palladium/production/s3fs-public/atoms/files/mcs-2019-cesiu.pdf (accessed 2022-08-05).
(12) Mineralogical Society of America. Pollucite $Cs(Si_2Al)O_6 \cdot nH_2O$. In *Handbook of Mineralogy*, Anthony, J. W., Bideaux, R. A., Bladh, K. W., Nichols, M. C. Eds.; Mineral Data Publishing, 2021.
(13) 3M. 3M™ Charge-Collection Solar Tape 3007. https://multimedia.3m.com/mws/media/756332O/3m-charge-collection-solar-tape-3007.pdf (accessed 2022-04-07).
(14) polymerdatabase.com. *Poly(butyl acrylate)*; polymerdatabase.com, 2022. https://www.polymerdatabase.com/polymers/polybutylacrylate.html (accessed 2022-08-15).
(15) Petrie, E. M. *Acrylic Pressure-Sensitive Adhesives (PSAs): A Comprehensive Guide*. adhesives.specialchem.com, 2022. https://adhesives.specialchem.com/selection-guide/acrylic-pressure-sensitive-adhesives-ingredient-formulation-tips?src=art-cnx (accessed 2022-08-15).
(16) Jones, D. *Factors affecting the selection and performance of silicone release coatings*; 2000008913; 2021. https://www.dow.com/documents/en-us/tech-art/25/25-87/25-877-01-factors-affecting-the-selection-and-performance-of-silicone-release-coatings.pdf (accessed 2022-07-26).
(17) polymerdatabase.com. *Poly(ethylene terephthalate)*; polymerdatabase.com, 2022. https://polymerdatabase.com/polymers/polyethyleneterephthalate.html (accessed 2022-07-26).
(18) Hischier, R.; Hellweg, S.; Capello, C.; Primas, A. Establishing Life Cycle Inventories of Chemicals Based on Differing Data Availability. *Int. J. Life Cycle Assess.* **2005**, *10* (1), 59–67. DOI: 10.1065/lca2004.10.181.7.
(19) Chemiepark Gendorf. *Umwelterklärung 2021*; 2021. https://www.infraserv.gendorf.de/-/media/Internet/infraserv_gendorf_de/Files/Broschueren/Unternehmen/DE/2021_Gendorf_Chemiepark_Umwelterklrung.ashx (accessed 2022-10-12).

(20) Department of Energy. *Determining Electric Motor Load And Efficiency*; DOE/GO-10097-517; Department of Energy, 2014. https://www.energy.gov/sites/prod/files/2014/04/f15/10097517.pdf (accessed 2021-03-27).
(21) van der Hulst, M. K.; Huijbregts, M. A. J.; van Loon, N.; Theelen, M.; Kootstra, L.; Bergesen, J. D.; Hauck, M. A systematic approach to assess the environmental impact of emerging technologies: A case study for the GHG footprint of CIGS solar photovoltaic laminate. *J. Ind. Ecol.* **2020**, *24* (6), 1234–1249. DOI: 10.1111/jiec.13027.
(22) Jungbluth, N.; Stucki, M.; Flury, K.; Frischknecht, R.; Büsser, S. *Life Cycle Inventories of Photovoltaics - Version: 2012*; ESU-services Ltd., 2012. https://esu-services.ch/data/public-lci-reports/ (accessed 2022-08-31).
(23) ITRPV. *International Technology Roadmap for Photovoltaics (ITRPV) - 2022 Results*; VDMA e. V., Frankfurt am Main, Germany, 2023. https://www.vdma.org/international-technology-roadmap-photovoltaic.
(24) Coletti, G.; Luxembourg, S. L.; Geerligs, L. J.; Rosca, V.; Burgers, A. R.; Wu, Y.; Okel, L.; Kloos, M.; Danzl, F. J. K.; Najafi, M.; et al. Bifacial Four-Terminal Perovskite/Silicon Tandem Solar Cells and Modules. *ACS Energy Lett.* **2020**, *5* (5), 1676–1680. DOI: 10.1021/acsenergylett.0c00682.
(25) Latunussa, C. E. L.; Ardente, F.; Blengini, G. A.; Mancini, L. Life Cycle Assessment of an innovative recycling process for crystalline silicon photovoltaic panels. *Sol. Energy Mater. Sol. Cells* **2016**, *156*, 101–111. DOI: 10.1016/j.solmat.2016.03.020.
(26) Mankins, J. C. *Technology Readiness Levels - A White Paper*. NASA, 1995. https://aiaa.kavi.com/apps/group_public/download.php/2212/TRLs_MankinsPaper_1995.pdf (accessed 2021-08-03).
(27) Moni, S. M.; Mahmud, R.; High, K.; Carbajales-Dale, M. Life cycle assessment of emerging technologies: A review. *J. Ind. Ecol.* **2020**, *24* (1), 52–63. DOI: 10.1111/jiec.12965.
(28) Fraunhofer ISE. *Photovoltaics Report - 6 December 2022*; Fraunhofer ISE, Freiburg, Germany, 2022. https://www.ise.fraunhofer.de/content/dam/ise/de/documents/publications/studies/Photovoltaics-Report.pdf (accessed 2023-01-02).
(29) Oxford PV. *Tandem cell production*. Oxford Photovoltaics Ltd., 2022. https://www.oxfordpv.com/tandem-cell-production (accessed 2023-01-02).
(30) ITRPV. *International Technology Roadmap for Photovoltaics (ITRPV) - 2021 Results*; VDMA e. V., Frankfurt am Main, Germany, 2022. https://www.vdma.org/international-technology-roadmap-photovoltaic (accessed 2022-11-30).
(31) Frelp by Sun. *Commercial interest – Phases 3 and 4*. 2022. https://www.frelp.info/interesse-commerciale-fasi-3-e-4/ (accessed 2023-08-03).
(32) Chen, B.; Fei, C.; Chen, S.; Gu, H.; Xiao, X.; Huang, J. Recycling lead and transparent conductors from perovskite solar modules. *Nat. Commun.* **2021**, *12* (1). DOI: 10.1038/s41467-021-26121-1.
(33) Vidal, R.; Alberola-Borràs, J.-A.; Habisreutinger, S. N.; Gimeno-Molina, J.-L.; Moore, D. T.; Schloemer, T. H.; Mora-Seró, I.; Berry, J. J.; Luther, J. M. Assessing health and environmental impacts of solvents for producing perovskite solar cells. *Nat. Sustain.* **2021**, *4* (3), 277–285. DOI: 10.1038/s41893-020-00645-8.
(34) Kim, H.-S.; An, Y.-J.; Kwak, J. I.; Kim, H. J.; Jung, H. S.; Park, N.-G. Sustainable Green Process for Environmentally Viable Perovskite Solar Cells. *ACS Energy Lett.* **2022**, *7* (3), 1154–1177. DOI: 10.1021/acsenergylett.1c02836.
(35) Stolz, P.; Frischknecht, R.; Wambach, K.; Sinha, P.; Heath, G. *Life Cycle Assessment of Current Photovoltaic Module Recycling*; IEA PVPS Task 12, International Energy Agency Power Systems Programme, Report IEA-PVPS T12-13:2018; 2017. https://iea-pvps.org/key-topics/life-cycle-assesment-of-current-photovoltaic-module-recycling-by-task-12-2/ (accessed 2020-04-15).
(36) ecoinvent database (Version 3.9.1) [Cut-off system model]. ecoinvent. https://v391.ecoquery.ecoinvent.org/ (accessed 2023-04-06).

(37) Steubing, B.; de Koning, D. Making the use of scenarios in LCA easier: the superstructure approach. *Int. J. Life Cycle Assess.* **2021**, *26* (11), 2248–2262. DOI: 10.1007/s11367-021-01974-2.
(38) Sacchi, R.; Terlouw, T.; Siala, K.; Dirnaichner, A.; Bauer, C.; Cox, B.; Mutel, C.; Daioglou, V.; Luderer, G. PRospective EnvironMental Impact asSEment (premise): A streamlined approach to producing databases for prospective life cycle assessment using integrated assessment models. *Renew. Sustain. Energy Rev.* **2022**, *160*, 112311. DOI: 10.1016/j.rser.2022.112311.
(39) Stehfest, E.; van Vuuren, D.; Kram, T.; Bouwman, L.; Alkemade, R.; Bakkenes, M.; Biemans, H.; Bouwman, A.; den Elzen, M.; Janse, J.; et al. *Integrated Assessment of Global Environmental Change with IMAGE 3.0 Model description and policy applications*; PBL Netherlands Environmental Assessment Agency, The Hague, The Netherlands, 2014. https://www.pbl.nl/sites/default/files/downloads/pbl-2014-integrated_assessment_of_global_environmental_change_with_image30_735.pdf (accessed 2020-12-17).
(40) O'Neill, B. C.; Kriegler, E.; Riahi, K.; Ebi, K. L.; Hallegatte, S.; Carter, T. R.; Mathur, R.; van Vuuren, D. P. A new scenario framework for climate change research: the concept of shared socioeconomic pathways. *Clim. Change* **2014**, *122* (3), 387–400. DOI: 10.1007/s10584-013-0905-2.
(41) van Vuuren, D. P.; Edmonds, J.; Kainuma, M.; Riahi, K.; Thomson, A.; Hibbard, K.; Hurtt, G. C.; Kram, T.; Krey, V.; Lamarque, J.-F.; et al. The representative concentration pathways: an overview. *Clim. Change* **2011**, *109* (1), 5–31. DOI: 10.1007/s10584-011-0148-z.
(42) Šimaitis, J.; Allen, S.; Vagg, C. Are future recycling benefits misleading? Prospective life cycle assessment of lithium-ion batteries. *J. Ind. Ecol.* **2023**. DOI: 10.1111/jiec.13413.
(43) Myhre, G.; Shindell, D.; Bréon, F.-M.; Collins, W.; Fuglestvedt, J.; Huang, J.; Koch, D.; Lamarque, J.-F.; Lee, D.; Mendoza, B.; et al. Anthropogenic and Natural Radiative Forcing. In *Climate Change 2013: The Physical Science Basis. Contribution of Working Group I to the Fifth Assessment Report of the Intergovernmental Panel on Climate Change*, Stocker, T. F., D. Qin, G.-K. Plattner, M. Tignor, S.K. Allen, J. Boschung, A. Nauels, Y. Xia, V. Bex and P.M. Midgley Ed.; Cambridge University Press, 2013.
(44) Muñoz, I.; Schmidt, J. H. Methane oxidation, biogenic carbon, and the IPCC's emission metrics. Proposal for a consistent greenhouse-gas accounting. *Int. J. Life Cycle Assess.* **2016**, *21* (8), 1069–1075. DOI: 10.1007/s11367-016-1091-z.
(45) Sand, M.; Skeie, R. B.; Sandstad, M.; Krishnan, S.; Myhre, G.; Bryant, H.; Derwent, R.; Hauglustaine, D.; Paulot, F.; Prather, M.; et al. A multi-model assessment of the Global Warming Potential of hydrogen. *Commun. Earth Environ.* **2023**, *4* (1). DOI: 10.1038/s43247-023-00857-8.
(46) Huijbregts, M. A. J.; Steinmann, Z. J. N.; Elshout, P. M. F.; Stam, G.; Verones, F.; Vieira, M.; Zijp, M.; Hollander, A.; van Zelm, R. *ReCiPe 2016 v1.1 A harmonized life cycle impact assessment method at midpoint and endpoint level Report I: Characterization*; RIVM, Bilthoven, The Netherlands, 2017. https://www.rivm.nl/sites/default/files/2018-11/Report%20ReCiPe_Update_20171002_0.pdf (accessed 2019-12-12).
(47) Steubing, B.; de Koning, D.; Haas, A.; Mutel, C. L. The Activity Browser — An open source LCA software building on top of the brightway framework. *Softw. Impacts* **2020**, *3*, 100012. DOI: 10.1016/j.simpa.2019.100012.
(48) *Activity Browser*; GitHub, 2024. https://github.com/LCA-ActivityBrowser/activity-browser (accessed 2024-02-08).
